# Reaction-Diffusion Dynamics Simulations of Bimolecular Quenching in Solution

**DOI:** 10.1021/acs.jctc.5c02114

**Published:** 2026-03-17

**Authors:** Simon A. Liedtke, Martin Trulsson, Petter Persson

**Affiliations:** Division of Computational Chemistry, Department of Chemistry, 5193 Lund University, Box 124, SE-22100 Lund, Sweden

## Abstract

A computational method to simulate bimolecular quenching reactions using coarse-grained reaction-diffusion dynamics is presented and applied to the quenching of molecular photosensitizers in solution. The simulations describe photoinduced reactions involving explicit excited states of light-harvesting species together with intrinsic deactivation, as well as collision quenching from separate quencher species. The simulation methodology is applied to time-resolved quenching of light-harvesting Fe­(III) complexes in electron-donating solvents as a prototype system for reaction-diffusion dynamics of experimental interest over a wide range of quencher concentrations. The results show clear signatures for the transition from classical diffusion-limited Stern–Volmer dynamics to close-contact quencher-photosensitizer interactions at high quencher concentrations, and the simulations are used to elucidate physically realistic photosensitizer-quenching collision interaction parameters for photoinduced dynamics beyond the classical Stern–Volmer model. The simulation method provides the means to directly model and analyze system kinetics and dynamics beyond standard theoretical equations, opening up significant opportunities to simulate a broad range of reactions in solutions.

## Graphical abstract

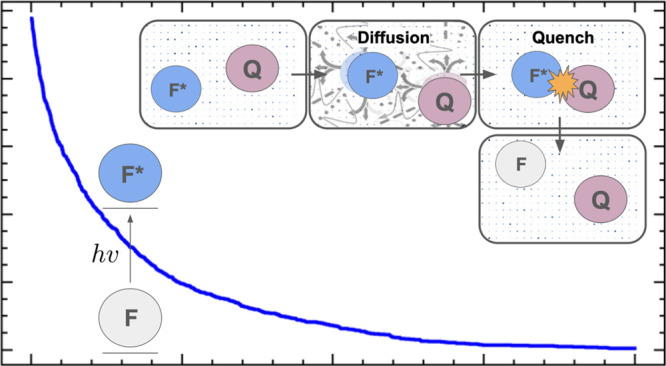

## 1 Introduction

Bimolecular quenching reactions are critical for photocatalysis and solar-fuel energy harvesting.^1–3^ Consequently, theoretical models and efficient methods of simulation that can shed light on these processes are highly desired for a fundamental understanding of the reaction dynamics and kinetics. However, the unique kinetics and dynamics involving photoredox processes make bimolecular Electron-Transfer (ET) mechanisms^4^ particularly difficult to simulate in solution.^5,6^ This is especially the case if explicit atomistic and quantum mechanical aspects of electron transfer and charge transfer are to be taken into account.^7,8^ This work focuses on a coarse-grained approach that enables the simulation of thousands of molecules while still including molecular parameters that are both physically and chemically relevant.

The Stern–Volmer (SV) model is the standard theoretical approach for describing bimolecular quenching kinetics^9,10^ with a theoretical rate constant that only depends on the radius and diffusion of the involved species, though one can also fit the fluorescence decay and effectively include all other unaccounted variables into the fitted rate. It should be noted that if the theory does not describe the underlying reaction diffusion dynamics properly, then the extracted rate lacks physical meaning. Evaluating systems via simulation, past the point at which the SV description is valid, in particular involving high quencher concentrations and/or nonsingle-exponential fitting, is thus especially relevant to both theory and experiment.

In this work, we aim to examine the diffusional reaction dynamics of bimolecular quenching processes across quenching concentration regimes and deviations from the classical Stern–Volmer model. The goal is to provide insight into fundamental system behavior for better control and understanding of charge recombination (CR) and charge separation (CS) processes at the heart of the bimolecular quenching reaction. Specifically, we utilize Langevin Dynamics with physically relevant coarse-grained molecular parameters and implicit solvent viscosity in order to simulate systems of interest.

There has been some work done examining the kinetics and dynamics of bimolecular quenching processes,^11–13^ but there has not been an examination of classical SV regime breakdown in simulation and introduction of experimentally relevant units and model parameters in such a simulation framework. We provide a general framework upon which to expand for future bimolecular quenching simulations, which can be compared to experimental fluorescence measurement of kinetic and dynamic system properties.

Additionally, we will focus on systems where the fluorophore-quencher pairs, (F) and (Q), act as electron donor and acceptor pairs. The quenching event is seen as near instantaneous, provided *k*_CR_ ≫ *k*_CS_ (with *k*_CS_ and *k*_CR_ denoting the rates of charge separation and charge recombination, respectively). Here, charge separation is the rate-determining step and, hence, effectively determines the close contact quenching rate. This is the case because a CS event occurs when the excited fluorophore (F*) initially interacts with a quencher (Q), and if the charge separated species are unable to diffuse away fast enough before the CR event takes place, the molecules return to their respective ground states. Such fast CR inhibits cage escape and photoproduct formation, which allows for the system to be modeled as a pseudo-first-order kinetic model. The decay/quenching rate of F* is proportional to the concentration of both quencher and F*. We further assume that the quencher can be reused, i.e., [Q] is constant (brackets indicate concentrations), given the short lifetime of any intermediate species produced during the quenching process.

Assuming instantaneous quenching, as described above, we have the following kinetic reaction scheme (set of reactions in eq 1) for a pseudo-first-order bimolecular quenching process (also illustrated in Figure 1):

Excitation{(ia)F+hv→δF*(ib)FQ+hv→δF*QIntrinsicdeactivation{(iia)F*→koF(iib)F*Q→koFQ

Diffusion{(iiia)F*+Q⇌kdissoc.kassoc.F*Q(iiib)F+Q⇌kdissoc.kassoc.FQQuenching{(iva)F*Q→kCCFQ(ivb)F*+Q→kqF+Q,=(iiia+iva) 1

**Figure 1:**
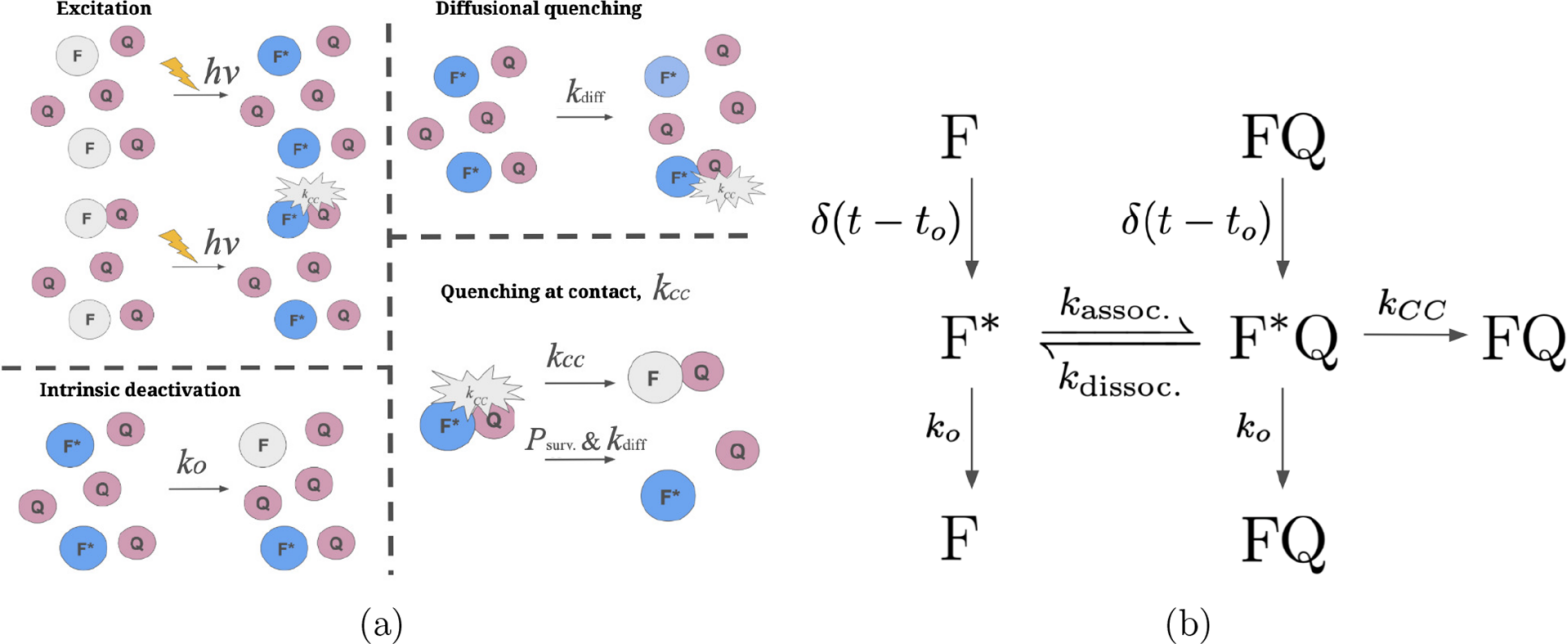
(a) Schematics of reactions in steps (ia)–(ivb). Quenchers (Q) are colored red, and fluorophores are blue (F*) if excited or gray in their ground state (F). (b) Kinetic diagram showing the kinetic pathways for excitation, diffusional association, quenching, and intrinsic deactivation.

For simplicity, we can assume that (ia) is a delta-pulsed, δ, excitation which excites all ground-state fluorophores at the given time of pulse. (ib) accounts for the same delta pulse but for fluorophores which already have a quencher as a neighbor within quenching distance at the time of excitation. (iia-b) is the intrinsic excited-state deactivation which occurs at the rate *k*_*o*_ (giving an intrinsic de-excitation lifetime of τ_0_ = 1/*k*_0_ in the absence of a quencher). (iiia) Diffusion of excited-state fluorophore and quencher coming into the vicinity for quenching to take place, and where we denote associative and dissociative diffusion contributions by *k*_assoc._ and *k*_dissoc._, respectively. (iiib) Diffusion of ground-state fluorophore and quencher. (iva) The excited-state fluorophore is deactivated by the quencher within close-contact distance at a rate of *k*_CC_ (which incorporates the combined rates of *k*_CS_ and *k*_CR_, assuming *k*_CR_ ≫ *k*_CS_ and CS is rate-determining). (ivb) *k*_*q*_ incorporates both the diffusional and the quenching rates from (iiia) and (iva) utilized in an SV description.

Over time, the excited-state population will often behave according to the pseudo-first SV order model (eqs 3 and 4):

$$\frac{d[F^{*}]}{dt}=\delta (t-t_{o})[F]-(k_{o}+k_{q}[Q])[F^{*}]$$ 3

$$\left[F^{*}(t)\right]=\left[F^{*}(0)\right]\exp[-t(k_{o}+k_{q}[Q])]$$ 4

where *k*_*o*_ is given in units of time^–1^ and *k*_*q*_ in (conc. × time)^−1^.

Though it will not be fitted or theoretically evaluated in our findings, this can be modeled as the following rate equation^10,14^ (eq 5), in rate-limiting-step form:

$$k_{q}=\frac{k_{\mathrm{diff}}k_{\mathrm{CC}}}{k_{\mathrm{diff}}+k_{\mathrm{CC}}}$$ 5

$$\mathrm{With},\;\lim_{k_{\mathrm{CC}}\to \infty }\;k_{q}\approx k_{\mathrm{diff}}$$ 6

Here, *k*_CC_ → *∞* implies instantaneous quenching (eq 6). This model assumes that all fluorophores are excited at a time of excitation (*t*_*o*_), shown with the δ pulse, and also that the diffusional flux, *k*_diff_, is the observed combination of *k*_assoc._ and *k*_dissoc._. In the context of our paper, the [F*] population is treated as 0 until the time of excitation (*t*_*o*_).

In practice, the δ pulse (reaction (ia) in the kinetics scheme above) is not totally efficient in experiment, such that not all ground-state fluorophores are excited by light, and there is additionally a probability of the fluorophore being quenched immediately upon excitation. For simplicity, we treat all fluorophores as excited in our simulations. This contrasts with experimental conditions where only a fraction of fluorophore species are excited; however, our theoretical treatment is for statistical purposes of fluorescence monitoring and should not qualitatively alter the experimental comparison at such low fluorophore concentration and distribution. In reality, the reaction dynamics may be complicated further with a distance-dependent quenching rate *k*_rxn_(*r*), accounting for distance-dependent electron transfer,^7^ where *r* refers to the center-to-center distance between two interacting molecules. Evidently, there is much more to account for beyond the classical Stern–Volmer model and room for simulations to shed light on more realistic reaction dynamics.

In the classical SV description^9,10,14^ of quenching kinetics, a *k*_*q*_ value can be extracted at a given quencher concentration [Q]. When purely theoretical treatment of diffusional quenching in the Stern–Volmer regime is used for experimental comparison, *k*_*q*_ is given by *k*_*q*_ = 4π*RD*_*o*_ *N*_A_. Here, *R* is the sum of the quencher (*R*_Q_) and fluorophore (*R*_F_) radii, *D*_*o*_ is the mutual diffusion coefficient $D_{o}=\frac{k_{B}T}{6\pi \eta R_{Q}}+\frac{k_{B}T}{6\pi \eta R_{F}}$ , and *N*_A_ is Avogadro’s number.^14^ Classical SV kinetics for the excited fluorophore then follows a single exponential quencher-dependent fluorescence decay. Note that *k*_CC_ is not explicitly accounted for, in that there is assumed immediate quenching upon contact in the SV model. From now on, *t*_0_ = 0, for notational convenience.

The experimentally relevant fluorescence intensity *I* (eq 7) can be obtained as

$$I=k_{f}\int_{0}^{\infty }\left[F^{*}(t)\right]dt$$ 7

where *k*_*f*_ is the radiative decay rate.

From the SV theory,^9,10^ knowing that $I=\frac{k_{f}\left[F^{*}(0)\right]}{k_{o}+k_{q}[Q]}$ and $I_{o}=\frac{k_{f}\left[F^{*}(0)\right]}{k_{o}}$ (where *I*_0_ is the fluorescence intensity in the absence of the added quencher), we now have the classical SV expression (eq 8) relating the fluorescence intensity to the quencher concentration:

$$\frac{I_{o}}{I}=1+\frac{k_{q}[Q]}{k_{o}}$$ 8

Several more advanced theories have been proposed by Smoluchowski,^15^ Collins–Kimball,^16^ Szabo,^17^ and others, which will be discussed in the Section 3 when comparing to the simulated data. These attempts incorporate a more detailed description of diffusion and reaction dynamics. A particularly valuable innovation, compared to classical SV, used in all of these models is treatment of the rate of reaction *k*_*q*_ in a time-dependent manner, *k*(*t*), such that the diffusional rate of reaction may be expressed in a non-Markovian fashion, with the time-instantaneous rate of quenching dependent upon the preceding time-sequence of events.^14^ This is not to express that the actual rate is changing over time but rather that the distribution and diffusion change over time and can be incorporated into an overall time-dependent rate. The more detailed dynamics may include molecular preaggregation, a transient diffusion time scale, and inefficient quenching. These effects can be directly simulated and evaluated for theoretical and experimental comparison.

In this work, we implement a coarse-grained Molecular Dynamics (MD) simulation approach to model photoinduced reaction-dynamics processes. In particular, each molecule is treated as a sphere with a truncated and shifted Lennard–Jones (LJ) force field interaction (Weeks–Chandler–Andersen (WCA) force-field); additionally, the simulations are performed using a Langevin thermostat, which allows for accurate diffusion and viscosity damping to account for the implicit solvent. This enables the simulation of up to tens of thousands of molecules and the observation of relevant kinetics and dynamics, which are available for experimental comparison. This simulation method allows for the comparison of quenching dynamics at high concentrations which are not typically simulated, though they exhibit unique and interesting quenching dynamics.^8,18^ The selection of accurate coarse-grained force fields is important to study such systems, and including an attractive component or different repulsive strengths will drastically change the observed quenching dynamics. Hence, having two potentials, one with attraction and the other without, allows one to study the effect of short-range attractive interactions. The truncated soft-sphere potential (WCA) allows us to closely model hard-sphere interactions, without needing to deal with the discontinuity in force and potential of the hard-sphere potential.

Theoretical analysis and calculation of bimolecular reaction-diffusion dynamics may be solved by using a variety of methods, such as the numerical solution of partial differential equations (PDE) describing the relevant system. However, the PDE description looks at overall concentrations as apposed to monitoring individual particle dynamics, and diffusion is highly approximated. In comparison, MD provides the advantage of explicitly modeling diffusion and directly simulating the reactive behavior of individual particles in their modeled environment for relevant analysis.

Here, we simulate the quenching of Fe­(III) N-heterocyclic carbene (NHC) champion photosensitizer, Fe­(phtmeimb)_2_^+^ as a relevant reaction of interest,^19–23^ in the study of photoinduced bimolecular charge transfer processes. Particularly, the experimental results and system parameters in Rosemann et al.^21^ provide relevant opportunity for comparison with simulation, as the charge recombination rate (*k*_CR_ ≈ 0.2 ps^–1^) was shown to be much faster than that of charge separation (*k*_CS_ ≈ 0.05 ps^–1^) for the fluorophore-quencher pair Fe­(phtmeimb)_2_^+^ and triethylamine. Additionally, the coarse-grained simulation of these processes provides a base to incorporate results and insights from atomistic simulations of cage escape processes,^8^ as well as to provide opportunities to explore questions of current experimental interest regarding cage escape.^24–26^

## 2 Methods

This study is based on the implementation of a coarse-grained simulation approach for photoinduced reaction-diffusion bimolecular quenching reactions in solution. Our approach utilizes the Langevin Dynamics OVRVO Verlet algorithm^27–29^ (equivalent to OBABO and the Bussi–Parinello algorithm) for implementation of a stochastic thermostat which also accounts for implicit solvent viscosity. Both contact and distant-dependent quenching reactions are also implemented in the Molecular Dynamics framework, allowing for reaction diffusion simulation. Additionally, we have Periodic Boundary Conditions (PBC), a constant number of particles, and a constant volume. The resulting simulations sample the NVT (canonical) ensemble, and the statistical results may then be evaluated and compared to experimental values of interest.

Given the Langevin Equation, where *d* **W**(*t*) is a standard Wiener noise, we have the following dynamics:

dr(t)=v(t)dt 9a

$$dv(t)=\frac{f(r(t))}{m}dt-\gamma v(t)dt+\sqrt{\frac{2k_{B}T\gamma }{m}}dW(t)$$ 9b

Here, we have vectors **r**(*t*) for positions, vectors **v**(*t*) for velocities, and vector **f**(**r**(*t*)) for forces on all particles at time *t*. $\gamma =\frac{6\pi \eta R}{m}$ is the friction coefficient, and η is dynamic viscosity. *R* is the radius of the particle and *m* the particle mass. (Note that *R*, *m*, and γ are also vectors, as mass and radius may differ for each particle.)

The equations of motion (eqs 9a and 9b) are integrated using the Trotter splitting and Liouville operators (using *b* = 1 in the standard OVRVO algorithm).^27^ The integration proceeds as follows: Half-step integration of the velocities (eq 10a), updating the positions (eq 10b), calculating new forces based on the new positions (eq 10c), and then integrating the velocities one-half-step further (eq 10d).

Trotter Splitting and Liouville operators are used to give the standard OVRVO *w*/*b* = 1 ^27^

$${\tilde{v}}_{t+\Delta t}=\frac{f(r_{t})}{2m}\Delta t+\sqrt{c}v_{t}+\sqrt{k_{B}T/m}\sqrt{1-c}N_{1}$$ 10a

$$r_{t+\Delta t}=r_{t}+{\tilde{v}}_{t+\Delta t}\Delta t$$ 10b

$$\mathrm{New}\;\mathrm{Forces},\;f(r_{t+\Delta t}),\;\mathrm{updated}\;\mathrm{based}\;\mathrm{on}\;r_{t+\Delta t}$$ 10c

$$v_{t+\Delta t}=\sqrt{c}\frac{f(r_{t+\Delta t})}{2m}\Delta t+\sqrt{c}{\tilde{v}}_{t+\Delta t}+\sqrt{k_{B}T/m}\sqrt{1-c}N_{2}$$ 10d

Here, *c* = exp­(−γΔ*t*); and $N_{1}$ and $N_{2}$ are vectors of size three with zero-mean, unit variance, independent Gaussian random variables.

OVRVO when reduced to one Gaussian, by skipping the half-step, is equivalent to the stochastic dynamics algorithm used in Gromacs.^30^ The OVRVO scheme has advantages for path sampling and path-reweighting, but for the purposes of our simulations, one could also use other standard Langevin integrators.

The Force Field (FF) used is either the traditional Lennard–Jones (LJ) potential^31^ (eq 11) or the Weeks–Chandler–Andersen (WCA) potential^32^ (eq 12), see Figure 2a for visualization. The WCA is a truncated and shifted Lennard–Jones potential, leading to a repulsive soft potential where the potential is 0 past the bottom of the LJ well (at *r* = 2^1/6^σ). With the potentials *U*(*r*_*ij*_), given the center-to-center distance *r*_*ij*_ between particles *i* and *j*, as follows:

$$U_{\mathrm{LJ}}(r_{ij})=4\varepsilon \left[{\left(\frac{\sigma }{r_{ij}}\right)}^{12}-{\left(\frac{\sigma }{r_{ij}}\right)}^{6}\right]$$ 11

and

$$U_{\mathrm{WCA}}(r_{ij})=\{\begin{array}{ll} 4\varepsilon \left[{\left(\frac{\sigma }{r_{ij}}\right)}^{12}-{\left(\frac{\sigma }{r_{ij}}\right)}^{6}\right]+\varepsilon , & r_{ij}\leq 2^{1/6}\sigma \\ 0, & r_{ij}>2^{1/6}\sigma \end{array}$$ 12

**Figure 2:**
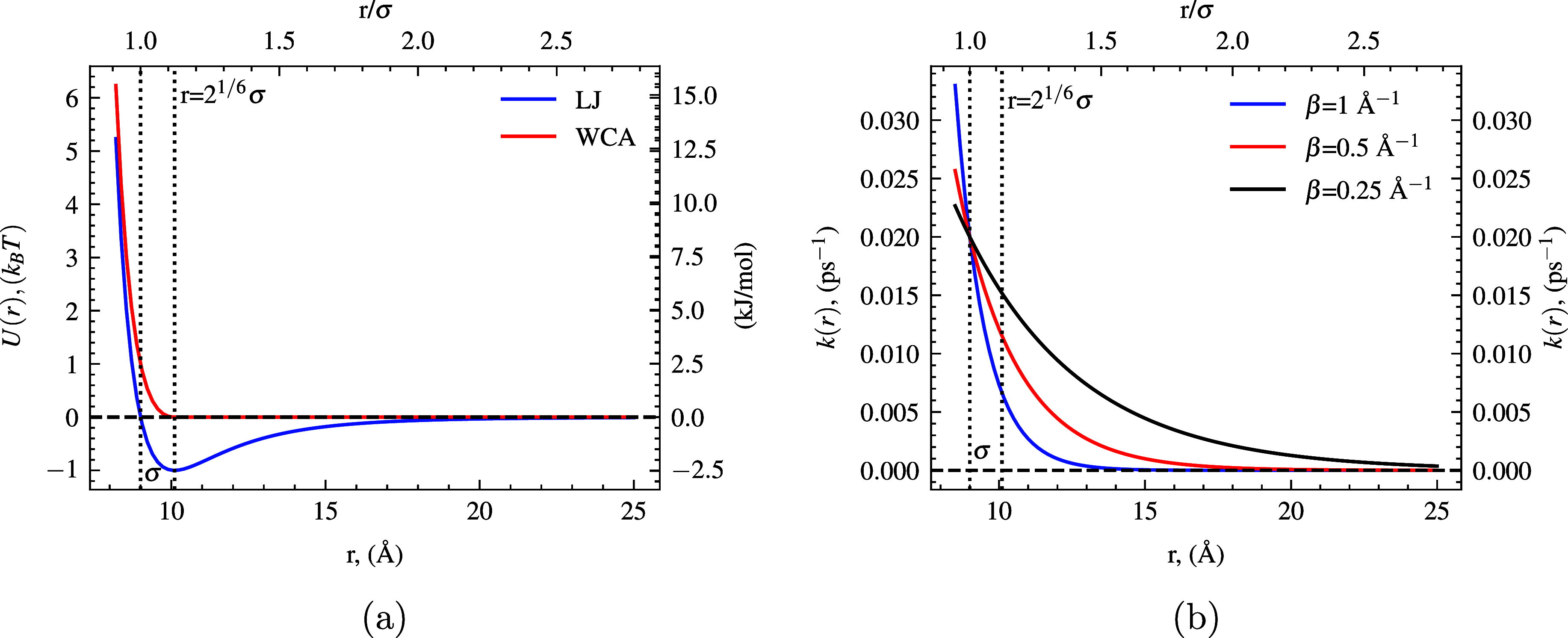
(a) Interaction potentials of the LJ and WCA for the Q–F interaction versus distance. (b) Distance-dependent quenching rate *k*_rxn_^*B*^(*r*) = *k*_CC_exp­(−β­(*r* – *r*_*o*_)) is shown for various β values. Vertical dotted lines show the contact distance (σ = *R*_Q_ + *R*_F_ = 9 Å) and the LJ minimum (2^1/6^σ).

See Figure 2a, where σ = (*R*_*i*_ + *R*_*j*_) is the mutual diameter of the particles, and ε encodes the interaction strength (both for the repulsive and attractive portion). We used the same ε for all pairs of species.

As one may intuitively conclude, when the full LJ is used (without truncation), there is a shorter fluorescence lifetime due to more average quencher neighbors at excitation and more attraction of nearby quenchers diffusing as well; this difference is most pronounced at intermediate concentrations and larger ε well depth. The choice of force field has significant impact on fluorescence intensity results, and thus, care needs to be taken when choosing attractive components in force fields and more atomistic-level detail. In our case, the simple WCA model serves as a suitable model to demonstrate reaction-diffusion quenching without attractive components affecting dynamics.

Additionally, ions in the experimental system and their Coulombic interactions may be explicitly included in the simulations; we account for this via the Wolf potential^33–35^ (eq 13) approximation (see SI for Coulomb force comparison). We therefore also performed simulations where anions were explicitly included with the same WCA and a Wolf potential to include Coulombic forces. According to the Wolf potential:

$$U_{\mathrm{Wolf}}(r_{ij})=\frac{1}{2}\Sigma_{i=1}^{N}\Sigma_{j\neq i}^{N}\frac{q_{i}q_{j}}{4\pi \varepsilon_{o}\varepsilon_{r}}\left[\frac{\mathrm{erfc}(\alpha r_{ij})}{r_{ij}}-\frac{\mathrm{erfc}(\alpha R_{c})}{R_{c}}\right]-\left[\frac{\mathrm{erfc}(\alpha R_{c})}{2R_{c}}+\frac{\alpha }{\sqrt{\pi }}\right]\Sigma_{i=1}^{N}\frac{q_{i}^{2}}{4\pi \varepsilon_{o}\varepsilon_{r}}$$ 13

where ε_*o*_ is the dielectric permittivity of vacuum, ε_*r*_ is the relative permittivity of the solution, *q*_*i*_ is the Coulomb charge, *R*_*c*_ is the finite cutoff radius (implemented as box length *L* divided by 2, *R*_*c*_ = *L*/2), and α = 2/*R*_*c*_ is used as the complementary error function damping parameter.

A quenching rate (*k*_CC_) is introduced for quenchers that are within the quenching distance of excited fluorophores. The continuous rate is implemented via conversion to a discrete probability of quenching per time step (see SI for details). A similar implementation can be seen in other work which examines macromolecular crowding and short-range caging on reaction rates.^36,37^

Thus, for the simple stepwise cutoff probability of reaction (eq 14), with *R*_react_ = 2^1/6^σ and σ = *R*_Q_ + *R*_F_, we have

$$k_{\mathrm{rxn}}^{A}(r)=k_{\mathrm{CC}}\Theta (R_{\mathrm{react}}-r)$$ 14

where Θ­(*x*) is the Heaviside step-function.

A distance-dependent ET-type efficiency (eq 15), visualized in Figure 2b, may also be introduced:^38^

$$k_{\mathrm{rxn}}^{B}(r)=k_{\mathrm{CC}}\exp(-\beta (r-r_{o}))$$ 15

Here, *k*_CC_ is treated as a free parameter that approximates a close-coupling Marcus-type ET rate, where *r*_*o*_ is the contact distance between reactants, and β is the distance-dependent coupling decay. This simplified form of a Marcus-type electron transfer uses a constant to represent the maximum quenching rate at the encounter distance, with distance-dependent decay screened by β.^39–42^ It has been reported for β to generally be ≈1 Å^–1^.^43^

Formally, we have, for a generic *k*_rxn_(*r*) and assuming *r* is fixed, the probability (eq 16) of a Q–F* pair quenching:

$$P_{\mathrm{quench}}(\delta t)=1-P_{\mathrm{surv}}(\delta t)=1-e^{-k_{\mathrm{rxn}}(r)\delta t}\approx k_{\mathrm{rxn}}(r)\delta t$$ 16

δ*t* is the time spent within the quenching distance. *P*_surv_(δ*t*) is the F* survival probability. The Taylor expansion approximation used for small δ*t* is sufficient in our simulations (see details in SI).

For implementation, a random number *u* (sampled uniformly between 0 and 1) is generated at each time step, Δ*t*, in which the quencher is within quenching distance, and the reaction takes place if *u* < *k*_rxn_(*r*)­Δ*t*. This probability of quenching at each time step represents inefficient quenching, averaged over orientational effects and distance dependence.

## 3 Results

### 3.1 Diffusion Dynamics

As a stepping-stone toward bimolecular quenching simulations, we first simulate regular solvation dynamics of a bimolecular system in solution at the coarse-grained level with a system comprising explicit fluorophores (F) and quenchers (Q) moving in an implicit solvent. The two compounds, Fe­(III) fluorophore complex (F) and TEA quencher (Q), were modeled as spheres of different sizes and masses, which approximate the represented compounds. A diameter of 13 Å and a mass of 750.1 g/mol were chosen to match the molecular size of the Fe­(III) complex, with 5 Å diameter and 101.19 g/mol mass used for TEA.^21^ The Acetonitrile solvent is described implicitly through its viscosity η_*f*_ and relative dielectric permittivity ε_*r*_ at room temperature, with η_*f*_ = 0.343 mPa s and ε_*r*_ of 37.5. In all our simulations, we kept the number of F constant at 10 mM and varied the number of quenchers (from 10 to 7000 mM), in order to simulate the broad range of relevant experimental conditions ranging from low to high quencher concentration (M = mol/dm^3^). This quencher concentration range was chosen to compare with the results of Rosemann et al.^21^ and to ensure we have equal or excess quenchers to fluorophores and minimal-to-no interference between fluorophores (the dilute 10 mM F population are distributed and not attracted to one another). The quencher concentration is not consumed in the reaction process and stays constant. A cubic periodic simulation box with a box length of 250 Å was used for all concentrations up to 2000 mM, and a box length of 150 Å was used for all higher concentrations. Simulation data shown are the aggregation of statistics for 10 such simulations each data point unless otherwise noted. The length/volume of the box determines the number of fluorophores and quenchers in the simulation, as the number of molecules added is based on the concentration. Equilibration was performed for 100 ps and monitored until the average number of neighbors of each fluorophore was steady. The encounter distance for step-function quenching probability was chosen as *R*_react_ = 2^1/6^σ (the LJ attractive well depth point).

For our specific system, we use a time step, Δ*t*, of 1 fs and a room temperature of *T* = 298 K. After 2 ps of simulation, all fluorophores are excited. An ε value of 1 *k*_B_ *T* (∼2.4777 kJ/mol at *T* = 298 K) was used both for the standard LJ and the WCA potential for all particle–particle interactions. The majority of simulations shown use the WCA potential in order to avoid influence of an attractive component; the ε in this case will simply act as the repulsive wall (“hardness”) of the soft-spheres. The sensitivity effects of adding LJ attraction, relevant in other experimental systems where aggregation (increasing the rate of quenching) may be studied, can be seen in Figures 6, 7, and the SI.

Table 1 lists diffusion, *D*_*o*_, and the corresponding average speed, ⟨|*v*|⟩, for the specific fluorophore and quencher as defined in our simulations. Also shown are characteristic times^14^ for Langevin dynamics relaxation and diffusion of fluorophore and quencher, with parameters taken from our simulations.

**Table 1:** Diffusion, Average Speed, and Characteristic Times^a^

	fluorophore	quencher
$D_{o}=\frac{k_{B}T}{6\pi \eta R}$	0.0979 Å^2^/ps	0.2546 Å^2^/ps
$\langle |v|\rangle =\sqrt{8k_{B}T/m\pi }$	0.917 Å/ps	2.498 Å/ps
$\tau_{\mathrm{relax}}=\frac{m}{6\pi \eta R}$	0.2964 ps	0.1039 ps
$\tau_{\mathrm{diff}}=\frac{R^{2}}{6D_{o}}$	71.922 ps	4.092 ps

a *T* = 298 K, η = 0.343 mPa s, and fluorophore/quencher characteristics are as described in the text.

The observed diffusion becomes concentration-dependent due to increased crowding at high packing fractions, as shown in Figure 3. In particular, diffusion (calculated as the diffusion coefficient, *D*, from MSD = ⟨|*r*(*t*) – *r*(0)|^2^⟩ = 6*Dt* decreases unphysically as a function of concentration, especially at quencher packing fractions (ϕ) significantly greater than 1%. This is predicted by, and our results closely follow, the Enskog theory of diffusion, where $D=D_{o}\frac{(1-\phi {)}^{3}}{1-\phi /2}$ . *D*_*o*_ is the Stokes–Einstein mutual diffusion coefficient using only η_ACN_. Furthermore, as the quencher concentration increases, the quencher effectively becomes the solvent. This leads to a model limitation that must be addressed in order to simulate experimentally accurate diffusion. To address this discrepancy, we adjust the viscosity using a mixing law that modifies the implicit viscosity according to packing fraction. In this way, the implicit viscosity contribution from the solvent will be replaced by the explicit viscosity of the quenchers, which theoretically replace the solvent volume. Calculated diffusion values (*D*), across quencher concentration, are shown in Figure 3.

**Figure 3:**
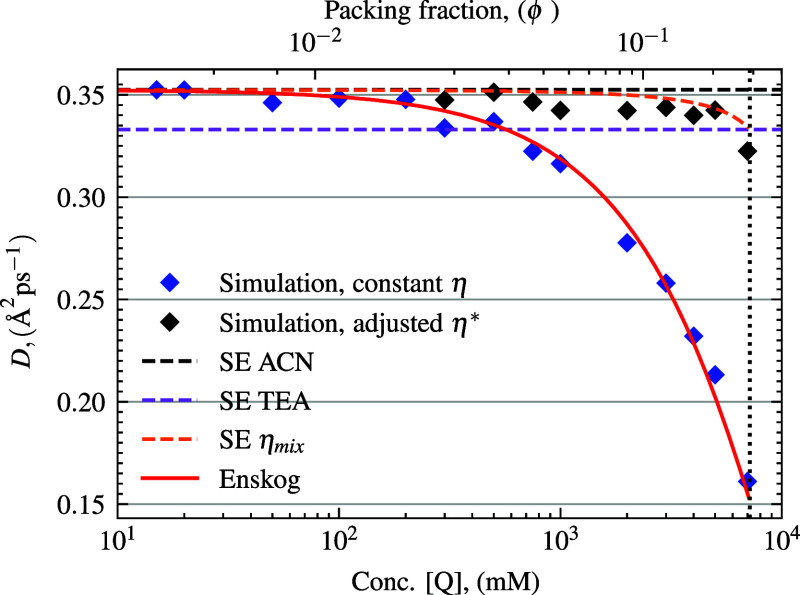
Simulation calculated diffusion coefficient, *D*, as a function of quencher concentration. Dashed lines show standard Stokes–Einstein (SE) diffusion theory values (for pure ACN, TEA, and the mixture). The solid red line showing Enskog theory which accounts for packing fraction. Simulation results are also plotted for constant η and adjusted η*. The dotted vertical line indicates the maximum concentration of quencher at 7.175 M. Shown on the semilog scale with concentration *x*-log.

A pure TEA solution with a density of 726 kg/m^3^ corresponds to a concentration of 7.175 M. Similarly, for pure ACN, a mass of 41.05 g/mol and density of 786 kg/m^3^ correspond to a maximum concentration of 19.147 M. We modify the viscosity of our simulations, such that the diffusion values will gradually approach the values which are expected in a pure TEA (quencher) solution. A simple Arrhenius mixing law^44^ for liquid mixtures was used: ln­(η_mix_) = Σ_*i*_ *x*_*i*_ ln­(η_*i*_). This gives mixed viscosity (η_mix_), which approaches that of TEA (η_TEA_ = 0.363 mPa ·s) from that of ACN (η_ACN_ = 0.343 mPa s), based on their molar fractions *x*_*i*_ (see SI for more details) such that ln­(η_mix_) = *x*_*TEA*_ ln­(η_TEA_) + *x*_ACN_ln­(η_ACN_). The calculated η_mix_ is used to solve the desired mutual diffusion, $D_{\mathrm{aim}}=k_{B}T\left(\frac{1}{6\pi R_{Q}}+\frac{1}{6\pi R_{F}}\right)\frac{1}{\eta_{\mathrm{mix}}}$ , at each quencher concentration [Q] (see Figure 3). This allows for the implementation of a modified viscosity value, η*, used in our simulations, where η* is calculated based on the reduced diffusion from the spheres according to Enskog diffusivity theory with the Carnahan–Starling expression for the Radial Distribution Function (RDF) at contact, $g(\sigma )=\frac{1-\phi /2}{(1-\phi {)}^{3}}$ , giving: $D=\frac{D_{o}}{g(\sigma )}=D_{o}\frac{(1-\phi {)}^{3}}{1-\phi /2}$ and $\eta^{*}=k_{B}T\left(\frac{1}{6\pi R_{Q}}+\frac{1}{6\pi R_{F}}\right)\frac{(1-\phi {)}^{3}}{1-\phi /2}D_{\mathrm{aim}}^{-1}$ .^45^

We begin to see significant change in mutual diffusion coefficient (for the uncorrected viscosity system) at quencher concentrations over 200 mM, as the simulated diffusion is much lower than that of the theoretical SE solution. Thus, in the simulations, at concentrations over 200 mM, it is important to use a corrected implicit viscosity, accounting for packing fraction friction. We were able to keep diffusion within the desired range, as seen in Figure 3, after applying the described theory which inputs the new η* based on packing fraction, ϕ. The resulting quenching results were then also able to be compared with those of the uncorrected model.

### 3.2 Quenching Simulations

Next, we consider results from simulations that explicitly include light-excitation pulses and subsequent excited-state deactivation of the photosensitizer complex due to either intrinsic deactivation or bimolecular quenching processes. This is done with the perspective to both shed light on when theoretical quenching models work or break down and to give insight into experimental results. The fluorescence decay curves, their fits, and SV fluorescence intensity plots are described and evaluated across concentration regimes and various types of quenching efficiency. The excited-state fluorophore, [F*], population decay over time is measured in the simulations for evaluation. Figure 4 shows F* fluorescence decay curves as a function of time at various quencher concentrations.

**Figure 4:**
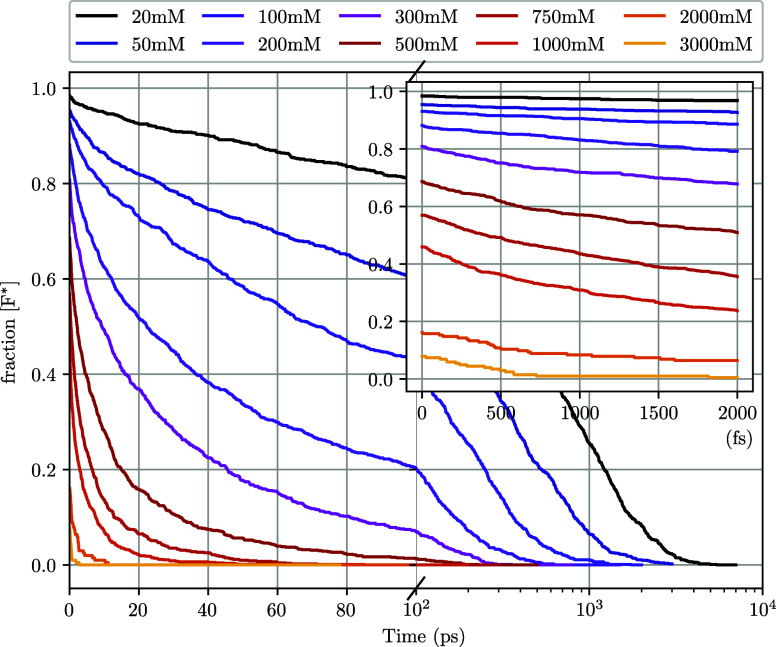
Excited-state fluorophore concentration ([F*]) as a function of time at various quencher concentrations. Simulations were performed using the WCA potential and diffusion-limited quenching (*k*_rxn_^*A*^(*r*) and *k*_CC_ → *∞*). Inset shows the initial portion of the de-excitation with clear static quenching occurring at the first time step.

Classical Stern–Volmer analysis of our simulation results begins to fall short at concentrations over 50 mM (see Figure 5 for fitting example), when we begin to see nonsingle exponential behavior, likely due to transient effects becoming more pronounced at these concentrations. Additionally, the static quenching contribution increases at higher concentrations due to having more quenching neighbors within the reaction distance. This effect can be clearly seen in the Figure 4 inset.

**Figure 5:**
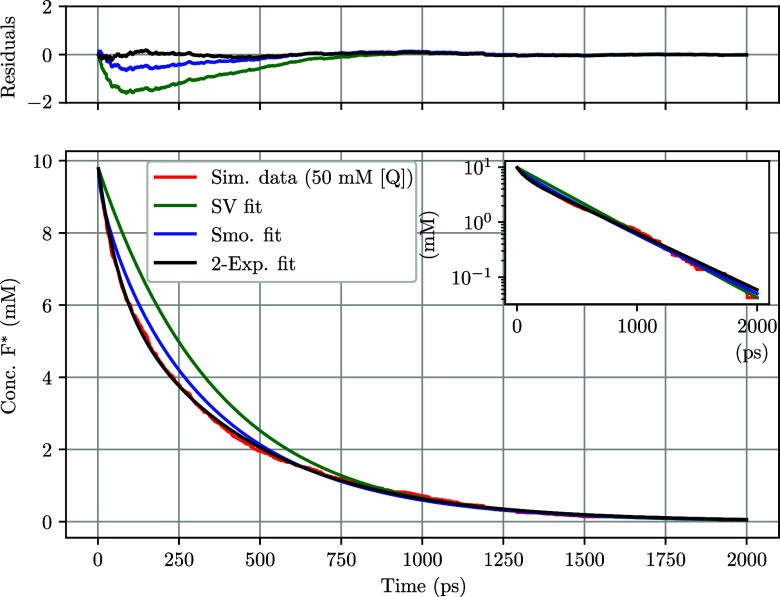
Excited fluorophore concentration as a function of time at 50 mM [Q]. The stepwise cutoff probability of reaction was used *k*_rxn_^*A*^(*r*) with *k*_CC_ → *∞* (diffusion limited). Time is shown starting from excitation time *t*_*o*_ set to 0, so the decay begins at 0. The data has been fitted with SV (SV fit), Smoluchowski fitting (Smo. fit), and fitting with a 2-exponential function (2-Exp. fit). Top panel shows fitting residuals, and the top-right inset shows semilog (*y*-log) scale.

Experimental data are often fitted phenomenologically by several exponentials, although it does not have a straightforward theoretical interpretation. *I* can be explicitly calculated by integrating the F* fluorescence decay curve over time (as according to eq 7), giving the *I*_*o*_/*I* – 1 data points. Explicit integrations were taken when full fluorescence decay curves were available for high concentrations. When long tails at low concentrations left the remaining fluorophores unquenched in our simulations, the decay curves were fitted to a two-exponential decay function with the tail extrapolated integrated numerically. The resulting $\frac{I_{o}}{I}-1$ results are plotted against concentration (log–log plots of $\frac{I_{o}}{I}-1$ against concentration will hereafter be referred to as SV-plots) and compared to the experimental data from Rosemann et al.^21^ (shown in plots as Exp. with blue squares) and theoretical intensities, as shown in Figure 6.

**Figure 6:**
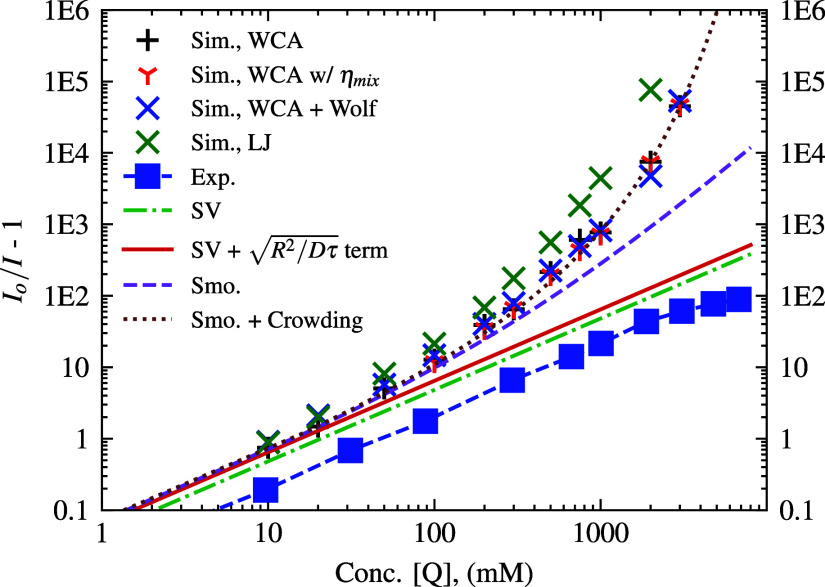
Log–log SV-plot of *I*_*o*_/*I* – 1 vs quencher concentration. Simulation results displayed for each concentration with WCA potentials, LJ potentials, and inclusion of Wolf potential and η_mix_ (all with *k*_rxn_^*A*^(*r*), using *k*_CC_ → *∞*). Theories shown include SV, Smoluchowski, SV with the extra term to match Smoluchowski at low concentrations, and Smoluchowski + Crowding, which incorporates the initial quench at excitation from neighbors. The experimental comparison is plotted with blue squares.

The mechanisms by which an excited fluorophore may return to the ground state, in our system, can be tracked and their relative contributions recorded, with these modes being: static quenching, diffusional quenching, and intrinsic deactivation (see Figure 7). Static quenching is the process in which the fluorophore and quencher are within quenching distance (via preaggregation or statistical positioning of the molecules) upon fluorophore excitation and near instant quenching occurs. This fraction is denoted as *P*_static_ = (F(0) – F­(0^+^))/F­(0). (0^+^ is used here to indicate that this is after close-contact static quenching upon excitation.) Note that this will depend on the rate, *k*_rxn_(*r*), and the time-window for which static quenching is defined. For the purpose of our simulations, we simply treat this time-window as 1 fs, although in practice this will be dependent upon the time-resolution of the experimental measurement device. Diffusional quenching occurs after a required mutual diffusion of the fluorophores and quenchers toward one another. This fraction is denoted as *P*_dynamic_. Intrinsic deactivation is when the excited-state fluorophore de-excites spontaneously according to the intrinsic deactivation rate, *k*_*o*_, without influence of any nearby quenchers. This fraction is denoted as *P*_intrinsic_.

**Figure 7:**
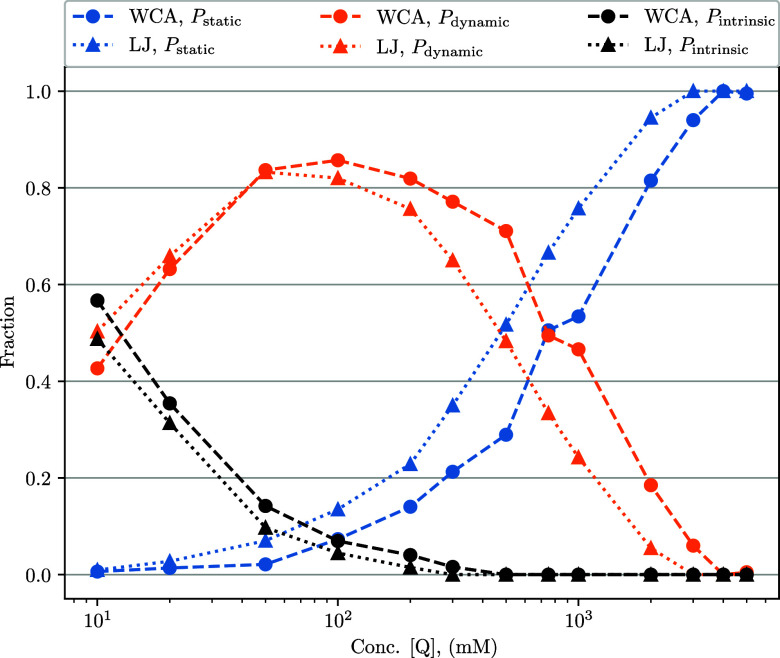
Fraction of different de-excitation contributions, *P*_static_, *P*_dynamic_, and *P*_intrinsic_, as a function of quencher concentration. Data correspond to simulations with either a WCA or a standard LJ potential (as denoted) with ε = 1*k*_B_T. With *k*_rxn_^*A*^(*r*), *k*_CC_ → *∞* in both cases. Lines are guides for the eyes. Shown on the semilog scale with concentration *x*-log.

These three mechanisms all compete for the responsibility of fluorophore deactivation, and their relative significance will vary in influence depending on concentration and the relevant rates. Thus, across the full lifetime of the excited state, the following conservation applies: *P*_static_ + *P*_dynamic_ + *P*_intrinsic_ = 1. As seen in Figure 7, the static portion (*P*_static_) of quenching increases with increasing concentration. This gives insight into experimental concentration limits for measuring fluorescence intensity when survival fraction is very low, although it is important to note the data in Figure 7 are evaluated at an infinite rate of quenching upon contact (*k*_CC_ → *∞*). This also indicates that the high-concentration *I*_*o*_/*I* values at this infinite rate, as shown in Figure 6, would not be accessible in experiment (due to incredibly low survival fraction) though they are able to be evaluated in our simulations. The dynamic quenching contribution (*P*_dynamic_) reaches its peak near the 100 mM quencher, at which point it starts to be outcompeted by the static component. Finally, the intrinsically quenched portion (*P*_intrinsic_) steadily decreases with increasing concentration, as it is competing with both static and dynamic quenching, which are both increasing. The results in Figure 7 are shown only for *k*_CC_ → *∞*, see the SI for contributions at a larger range of *k*_CC_ values.

Note that the intrinsic deactivation is competitive with diffusion quenching because τ_*o*_ here is quite short, at 2 ns for this specific photosensitizer. Other photosensitizers, which have τ_*o*_ values in the range of hundreds of nanoseconds or longer, would see *P*_static_ and *P*_dynamic_ completely dominating the *P*_intrinsic_.

Figure 6 displays the key results when only the step-function quenching, *k*_rxn_^*A*^(*r*), is implemented with *k*_CC_ → *∞* implemented. We have clear signatures for the transition from classical diffusion-limited Stern–Volmer dynamics to close-contact quencher-fluorophore interactions at high quencher concentrations, as seen from the nonlinear $\frac{I_{o}}{I}-1$ deviations following the Smoluchowski curve, and yet steeper nonlinear deviation past 300 mM. The curves at lower concentration ranges are usually still able to be fit with a straight line. However, as can be clearly seen with the full range of simulated concentrations, there is strong upward sloping behavior (indicating faster quenching than predicted by classical SV) for which simple diffusion alone does not account for.

The nonlinear $\frac{I_{o}}{I}-1$ results are attributed to the well-known transient effects at short time scales,^40,46,47^ which play a much larger role as the concentration increases and thus the time scale of total quenching time also decreases. Additionally, an increasing “crowding” quenching component is observed as more fluorophores have more quencher neighbors, within reaction distance, on average upon excitation.

Moreover, as Figure 6 shows, there is no visible difference in the results by either adding the Wolf potential to account for Coulomb forces (where we explicitly include the PF_6_^–^ anions) or by accounting for the η_mix_ changing with increasing concentration, as the data points are essentially atop of one another. The system was also simulated with the LJ potential added for all fluorophore-quencher interactions (Q–Q and F–F interactions still used only the WCA potential) and clearly resulted in larger $\frac{I_{o}}{I}-1$ values seen than those of the WCA potential system, with this difference being gradually more drastic with increasing quencher concentration. This is to be expected, with the attractive component of the LJ potential increasing associativity of the fluorophore and quencher both in the static and dynamic quenching interactions, referring to the SI for the RDFs which make this visibly evident.

As noted, there was little-to-no difference observed in the *I*_*o*_/*I* curves when Coulombic forces were included via the Wolf potential, indicating that Coulombic effects on quenching dynamics may be safely ignored for the current system of study due to low ion concentrations and little effect in blocking quencher-fluorophore collisions. Therefore, the presented simulations do not include the anion and Coulombic force terms, unless otherwise noted.

Including a decreasing (concentration-dependent) diffusion, according to Enskog theory, into the theoretical Smoluchowski^14,15^ equation (eq 19) for the *I*_*o*_/*I* – 1 graphs has a slight decrease in the theoretical *I*_*o*_/*I* – 1 magnitude at high concentration. However, it can be seen that accounting for a corrected diffusion (as we do in Figure 3) has little impact on the simulated SV-plot; see Figures 6 and 8. This indicates there is a negligible effect from whatever diffusion differences may exist, as fluorophores are in close contact at high concentrations. At least in this system of study, by the point at which we would expect to see reduced diffusion from packing influence the quenching rate dynamics, the quenching is dominated by the number of neighbors and diffusion has little-to-no impact on observed fluorescence. This relationship is expected, though it is not necessarily always so directly offsetting.

**Figure 8:**
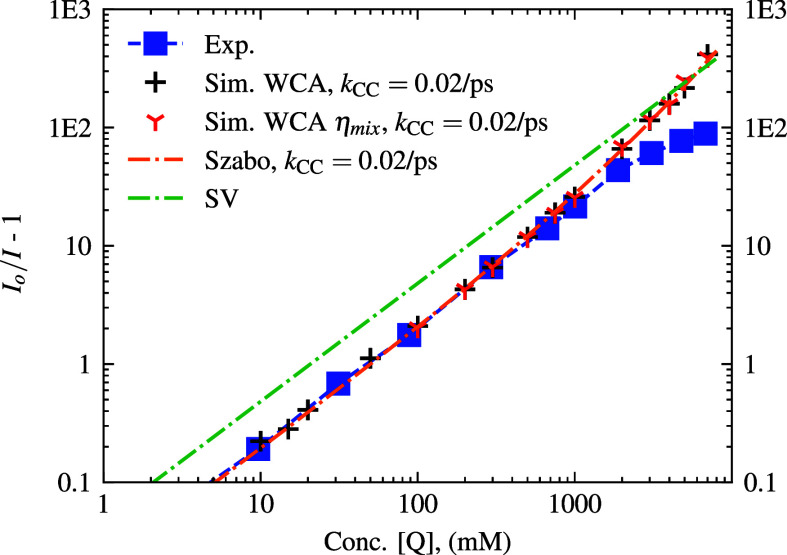
Log–log SV-plot of *I*_*o*_/*I* – 1 vs quencher concentration when inefficient quenching is implemented. Simulations were displayed for WCA potential with *k*_rxn_^*A*^(*r*) and *k*_CC_ = 0.02 ps^–1^. Data points for simulations with η_mix_ are also shown. Theoretical curves incorporating *k*_CC_ in the Szabo model are also included. The experimental comparison is plotted with blue squares.

In order to study inefficient quenching (as opposed to *k*_CC_ → *∞* instant reactions within reaction vicinity, *R*_react_) we implement, with standard WCA only simulations, the probability of quenching as a probability per time step derived from the rate of close contact quenching *k*_CC_ = 0.02 ps^–1^. As described above, this was chosen by fitting the 300 mM quencher concentration *I*_*o*_/*I* – 1 results in order to get a rough estimate of *k*_CC_ that would fit the experimental data. The influence of rate sensitivity has also been analyzed in the SI, which shows intensity differences according to theory (eqs 5 and 8) of *I*_0_/*I*. The chosen *k*_CC_ = 0.02 ps^–1^ is also comparable in magnitude to the CS/CR rates seen in the measurements from Rosemann et al.^21^ (*k*_CR_ ≈ 0.2 ps^–1^ and *k*_CS_ ≈ 0.05 ps^–1^). The chosen value of *k*_CC_ = 0.02 ps^–1^ was used for subsequent inefficient quenching simulations, see Figure 8 for the resulting SV-plot. This finite reaction probability provides a way to improve the agreement with experimentally observed quenching dynamics without resorting to nonphysical parameters such as diffusivity or contact radii of F* and Q molecules. The results from our simulations indicate that the majority of the points up to 1000 mM quencher concentration match the compared experimental values well, on the log–log scale (see Figure 8). However, the fluorescence intensity from the simulations indicates much faster quenching than what was observed experimentally. The Szabo model^17,48^ of inefficient bimolecular fluorescence quenching which is detailed later (eq 22) in the theory section fits well to our simulated data. Additionally, we again observed little-to-no difference in simulated results when η_mix_ was adjusted according to packing fraction, which we attribute to the relative quenching effect of a larger number of quencher neighbors dominating quenching by the time diffusion changes significantly enough to alter the dynamics.

Furthermore, in order to heuristically examine the consequences of inefficient quenching on the time of contact, we calculate the expected contact time (τ_contact,X%_) for *X*% probability of quenching: with the step function *k*_rxn_^*A*^(*r*) for Q–F* within *R*_react_, we have $\tau_{\mathrm{contact},X\%}=\frac{-\ln(1-X)}{k_{\mathrm{CC}}}$ , with results seen in Table 2.

**Table 2:** Expected Time of Contact (τ_contact,X%_) for a Given Rate of Quenching; Shown for the Rate of *k*_CC_ = 0.02 ps^–1^

τ_contact_	
prob. quenching (%)	expected time (ps)
25	14.384
50	34.657
90	115.129
99	230.259

The chosen experimental data exhibits a decreasing slope in *I*_*o*_/*I* – 1 fluorescence intensity ratio at high concentrations; this is the opposite of what one would expect from current theory and simulations. We implemented a method to reproduce this curvature via the interference of quenchers, such that a maximum number of quenchers can act on the excited fluorophore at one time. For example, if the maximum number of quenchers acting on an excited fluorophore is 2, and we have 5 quenchers within reaction distance at a time, then only 2 are evaluated with the probability to quench the excited fluorophore. This was implemented, in the simulations, with the max number of quenchers acting on a fluorophore to be either ≤1, ≤2, and ≤4; see Figure 9. The results for ≤2 appeared to best fit the experimental data. This indicates that interference effects can provide a hypothesis for the nonlinear decreasing slope curvature observed in the experimental *I*_*o*_/*I* – 1 data at high quencher concentrations. However, there may be other explanations for such a phenomenon.

**Figure 9:**
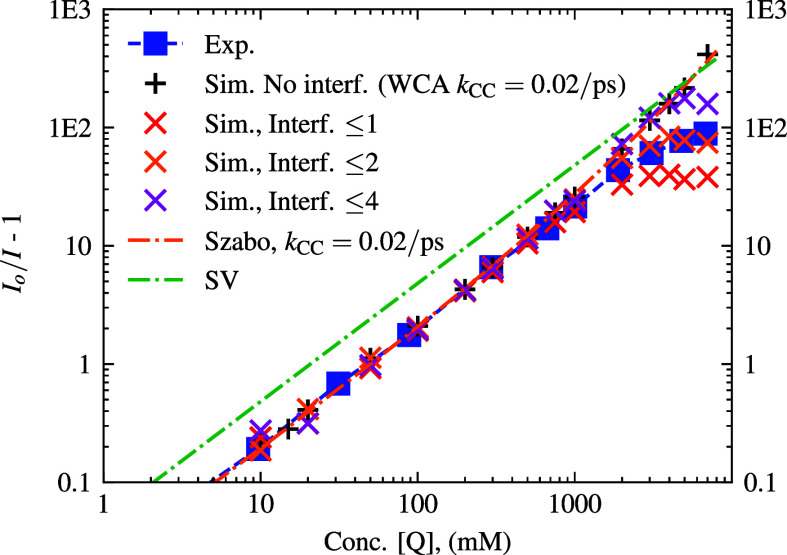
Log–log SV-plot of *I*_*o*_/*I* – 1 vs quencher concentration when inefficient quenching and quencher interference are implemented. All simulations have WCA potential with *k*_CC_ = 0.02 ps^–1^. The interference data assumes the quenchers interfere with each other’s quenching on the same fluorophore, and thus, we see a departure at higher concentrations when multiple neighbors may act on each fluorophore. Data shown for max quenchers acting on each fluorophore of 1, 2, and 4. The experimental comparison is plotted with blue squares.

The distance-dependent quenching rate, *k*_rxn_^*B*^(*r*), attempts to simulate a more physically realistic distance-dependent quenching that approximates more detailed electron-transfer models, as detailed above. *k*_rxn_^*B*^(*r*) = *k*_CC_exp­(−β­(*r* – *r*_*o*_)) is examined for β values of 0.25, 0.5, and 1 Å^–1^, see Figure 10b. The resulting curves give very similar results between β = 1 Å^–1^ and the simple step-function *k*_rxn_^*A*^(*r*) model, while the lower β values quench at a longer distance (quench faster) and thus have larger *I*_*o*_/*I* – 1 values. The quencher-fluorophore separation when quenching occurred is also displayed (see Figure 10a) and the histograms match the theoretical values based on the *k*_rxn_^*B*^(*r*) function and the corresponding RDF at each concentration. The theoretical quenching distance distribution calculation is obtained by numerically integrating the RDFs of our simulations with the quenching probability to give a theoretical distribution of quenching distances: $P(r)=\frac{g(r)r^{2}\exp(-\beta (r-r_{o}))}{\int_{0}^{\infty }g(r^{\prime })r^{\prime 2}\exp(-\beta (r^{\prime }-r_{o}))dr^{\prime }}$ .

**Figure 10:**
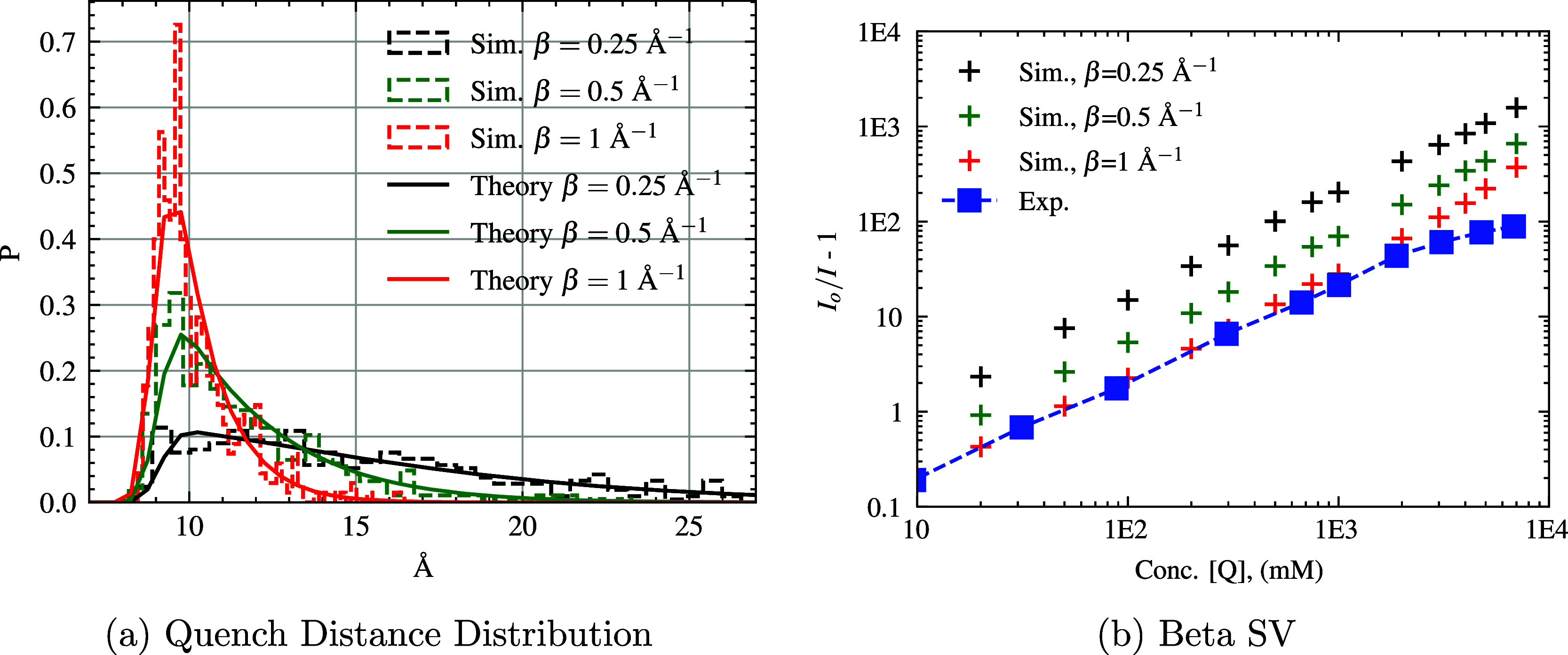
(a) The dashed histograms display the F*–Q quenching distances (center to center) observed in the simulation at 500 mM [Q] (using WCA potential, *k*_rxn_^*B*^(*r*), *k*_CC_ = 0.02 ps^–1^, and various β values). The solid lines show the theoretical result from the RDF of our simulations and the distant-dependent quenching probability, as detailed in the text. (b) Log–log SV-plot of *I*_*o*_/*I* – 1 vs quencher concentration, when the distance-dependent quenching rate is used (using WCA potential, *k*_rxn_^*B*^(*r*), *k*_CC_ = 0.02 ps^–1^, and various β values). The experimental comparison is plotted with blue squares.

We may extract the fluorescence lifetime values across concentration, in a straightforward manner (see Figure 11a), by calculating the time, where [F*] is the fraction $\frac{1}{e}$ of the initial concentration of [F*] (after immediate static quenching). The choice of $\frac{1}{e}$ for determining fluorescence lifetime is done in order to directly compare to the experimental lifetime metrics, based on the assumption of single-exponential decay which was used for the lifetime calculations in Rosemann et al.^21^ This single exponential assumption also gives a direct intensity comparison in the SV model when *I*_*o*_/*I* = τ_*o*_/τ, and it is a convenient metric to compare where the Smoluchowski decay equation reaches this point. The fitting of data with a two-exponential fit can also be evaluated as an intensity-weighted lifetime average,^49–51^ though the experimental system we are modeling has no direct metrics for comparison. The results largely correspond to what is described above for the fluorescence intensity *I*_*o*_/*I* – 1 graphs; however, a discrepancy in *I*_*o*_/*I* – 1 vs τ_*o*_/τ – 1 can be seen when plotted on the same graph, see Figure 11b. As the initial static component of fluorescence intensity, which greatly increases the *I*_*o*_/*I* – 1 values, it would suggest a faster rate of quenching even though it is the same data. This indicates a point of interest for experimentalists who may desire to identify initial static quenching by comparing *I*_*o*_/*I* – 1 and τ_*o*_/τ – 1 discrepancies, which are particularly clear at high concentrations.

**Figure 11:**
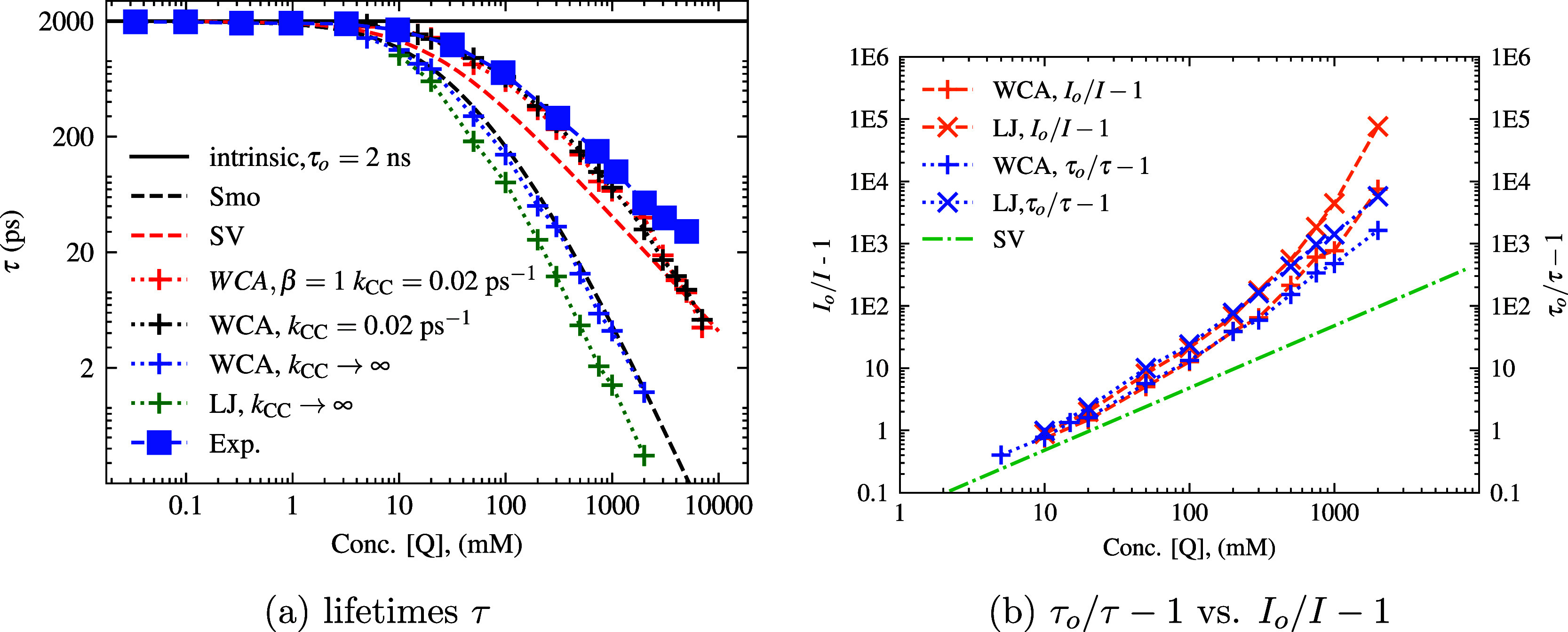
(a) Log–log plot of excited-state lifetimes vs quencher concentration. The excited-state lifetimes, τ, extracted from the simulations and theories as the time, where [F*] is the fraction $\frac{1}{e}$ of the initial concentration ([F*] = 10 mM). The experimental comparison is plotted with blue squares. (b) Log–log SV-plot comparing calculated τ_*o*_/τ – 1 vs *I*_*o*_/*I* – 1. Both graphs are plotted on the log–log scale.

The general simulation method is also able to provide the pair distribution F*–Q RDFs across different time points, *g*_F*Q_(*r*, *t*), (in comparison to a standard RDF at equilibrium), as the F* quenching is a dynamic process, and we have decreasing population of F*. See Figure 12 where we show time-dependent RDFs for a 500 mM quencher system with a step-function cutoff and *k*_CC_ = 0.02 ps^–1^. Figure 12 shows *g*_F*Q_(*r*, *t*) along with *g*_FQ_(*r*, *t*) (which is also changing due to changing F population) after aggregating statistics for 30 independent simulation runs. The right graph in Figure 12 shows these *g*(*r*, *t*) normalized by the fraction of either F­(*t*)/*N*_FF*_ or F*­(*t*)/*N*_FF*_ at that specific time (*N*_FF*_ being the total fluorophore population). The evolving nature of the average distance from excited-state fluorophore to quencher over time can be clearly seen.

**Figure 12:**
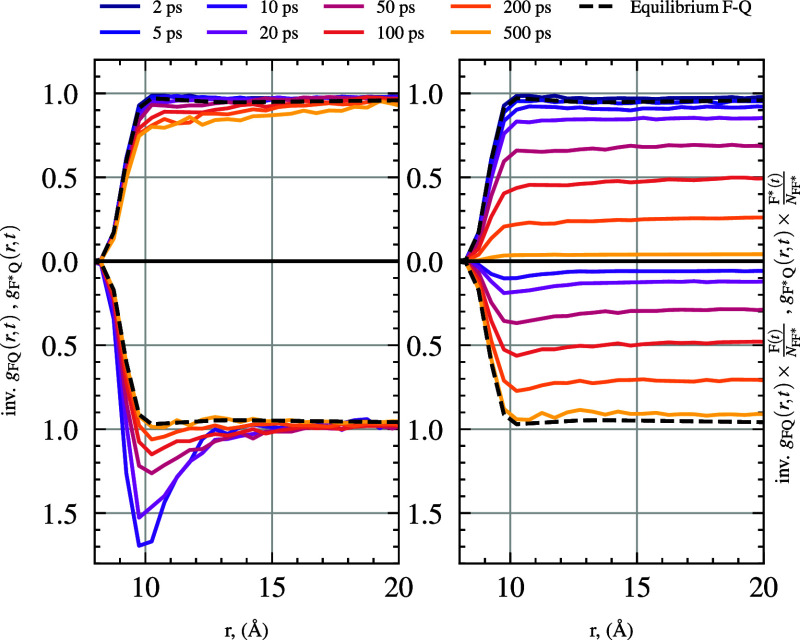
Top shows the RDF for F*–Q, bottom is the inverse RDF (reflected over the axis for comparison) for F–Q, Equilibrium F–Q (black dashed) is shown for reference in all plots. The right panel is normalized by multiplication of the population fraction of the total fluorophore (F­(*t*)/*N*_FF*_ or F*­(*t*)/*N*_FF*_). The RDFs were recorded for 500 mM [Q] and *k*_rxn_^*A*^(*r*) with *k*_CC_ = 0.02 ps^–1^.

A simple intuition for this phenomenon can be thought of as follows: the F* which have on average more nearby quenchers are quenched first through static or short-time diffusional quenching, then the remaining F*s are those that were not originally contributing to the close-separation portion of the RDF (and the mutual diffusion of F and Q toward each other is not sufficient to replace this change in distribution in the first RDF layer). The nonequilibrium state of F* concentration is induced by the pulse, and thus, the RDF may be monitored across time as the concentration of close quenchers varies out of equilibrium until the full set of reactions occur and the nearby molecules diffuse to the long-time distribution (or [F*] reduces to 0). These dynamically evolving RDFs, *g*_F*Q_(*r*, *t*) and *g*_FQ_(*r*, *t*), will naturally be highly dependent on *k*_rxn_(*r*) and quencher concentration.

These time-dependent RDF results are in agreement with existing bimolecular quenching theory,^52^ as described by Differential Encounter theory, where a time-dependent quenching rate, k­(t), is included to illustrate a transition from static, to nonstationary, to stationary quenching rates over time. [F*­(*t*)] = [F*(0)]­exp­(−*tk*_*o*_ – [Q]∫_0_^*t*^ *k*(*t′*)*dt′*), where *k*(*t*) = 4π∫_*r*=*r*_*o*__^*∞*^ *w*(*r*)*n*(*r*, *t*)*r* ^2^ *dr* and *w*(*r*) is the reaction probability (in principle the same as *k*_rxn_(*r*) we use in our model) and *n*(*r*, *t*) is the reactant pair distribution function (displayed in Figure 12 as *g*_F*Q_(*r*, *t*)). A PDE can be provided for the changing distribution function as follows: $\frac{\partial }{\partial t}n(r,t)=\hat{L}(r)n(r,t)-w(r)n(r,t)$ , with $\hat{L}(r)=\frac{1}{r^{2}}\frac{\partial }{\partial r}Dr^{2}$ $\exp(-v(r))\frac{\partial }{\partial r}\exp(+v(r))$ as the diffusion operator, and *v*(*r*) is the solvent (where *v*(*r*) = −log­(*g*(*r*)) and if we treat our solvent–solvent pair distribution function, *g*(*r*), as 1, then *v*(*r*) = 0).^52^

#### 3.2.1 Comparisons with Theoretical Models

Finally, we put our simulation results in the context of the established theoretical models used to describe quenching dynamics. A commonly used extension to the classical SV model, the Smoluchowski treatment of fluorescence quenching, includes an early time scale transient component which accounts for the nonsingle exponential fluorescence decay curves seen in many experiments and simulations.^10,14,15^ This leads to an additional $\sqrt{t}$ -component in the relaxation, important at short-times. The Smoluchowski time-dependent quenching rate coefficient with time-dependence, $k(t)=4\pi R_{\mathrm{FQ}}D_{o}N_{A}\left[1+\frac{R_{\mathrm{FQ}}}{\sqrt{\pi D_{o}t}}\right]$ , thus accounts for the non-Markovian transient effects which are seen in diffusional reaction dynamics.^14^ Furthermore, *k*_*q*_ is now expressed with the mutual diffusion coefficient *D*_*o*_ = *D*_F_ + *D*_Q_ and *R*_FQ_ = *R*_F_ + *R*_Q_ as the encounter distance (sum of radii) of F and Q. The diffusion for each molecule may be taken from the Stokes–Einstein equation: $D_{i}=\frac{k_{B}T}{6\pi \eta R_{i}}$ .

Recalling the earlier formula (eq 4) for classical SV, we restate it here as (eq 17):

$$[F^{*}(t)]=[F^{*}(0)]\exp(-t(k_{o}+[Q]k_{q})),\;\mathrm{where}\;\mathrm{theoretically}\;k_{q}=4\pi R_{\mathrm{FQ}}D_{o}N_{A}$$ 17

When the Smoluchowski time-dependent quenching rate, *k*(*t*), is incorporated into the decay function and integrated, we have the following well-known (eq 18) Smoluchowski decay function:

$$\left[F^{*}(t)\right]=\left[F^{*}(0)\right]\exp\left[-t\left(k_{o}+4\pi R_{\mathrm{FQ}}D_{o}N_{A}[Q]\left(1+\frac{2R_{\mathrm{FQ}}}{\sqrt{\pi D_{o}t}}\right)\right)\right]$$ 18

This gives the following *I*_*o*_/*I* relation:^14^

$$\frac{I_{o}}{I}=\frac{1+k_{d}\tau_{o}[Q]}{1-\frac{\sqrt{\pi }K[Q]}{\sqrt{\frac{1}{\tau_{o}}+k_{d}[Q]}}\exp\left(\frac{K^{2}[Q{]}^{2}}{\frac{1}{\tau_{o}}+k_{d}[Q]}\right)\mathrm{erfc}\left(\frac{K[Q]}{\sqrt{\frac{1}{\tau_{o}}+k_{d}[Q]}}\right)}$$ 19

where *k*_*d*_ = 4π*R*_FQ_ *D*_*o*_ *N*_A_ and $K=\frac{k_{d}R_{\mathrm{FQ}}}{\sqrt{\pi D_{o}}}$ .

The vertical-gap seen in the Stern–Volmer plots comparing Stern–Volmer theory against other theories such as Smoluchowski (eq 19) is from the constant additional factor at low concentrations $\approx \sqrt{\frac{R_{\mathrm{FQ}}^{2}}{D_{o}\tau_{o}}}$ . As can be shown, see Figure 6, this gap may be eliminated by adding the constant factor and we can modify the theoretical Stern–Volmer value to match the other theories at low concentrations, $\frac{I_{o}}{I}=1+\tau_{o}k_{d}[Q]\left(1+\sqrt{\frac{R_{\mathrm{FQ}}^{2}}{D_{o}\tau_{o}}}\right)$ .

The Collins–Kimball (CK) treatment is another common extension to the classical SV model and accounts for a *k*_act_ rate of quenching effectiveness which effectively adjusts the model radius in order to fit the data.^14^ This gives an effective radius as $R_{\mathrm{eff}}=\frac{R_{\mathrm{FQ}}k_{\mathrm{act}}}{k_{\mathrm{act}}+4\pi R_{\mathrm{FQ}}D_{o}}$ . Thus, *k*_act_ will also have units of fs^–1^. The *R*_eff_ value can subsequently be substituted into the Smoluchowski equation, giving the new CK $\frac{I_{o}}{I}-1$ value, by treating *k*_*d*_ = 4π*R*_eff_ *D*_*o*_ *N*_A_ and $K=\frac{k_{d}R_{\mathrm{eff}}}{\sqrt{\pi D_{o}}}$ and plugging these new values in eq 19.

Further additions to all these models may also be implemented, in particular immediate quenching by neighbors gives a modified Smoluchowski, accounting for the “crowding” quenching fraction, to better fit the *I*_*o*_/*I* results.^46,53,54^ At high enough concentrations, i.e., ≥1 M, on average, every fluorophore has over 1 quencher, see SI for figure of average neighbors at different concentrations. This is predicted from the Poisson probability distributions and seen from our own simulations. Assuming the distribution of molecules is random, *P*_*o*_ is the probability at least one Q molecule is located within *r*_*c*_ of F and contact distance *a*.

$$P_{o}=\Sigma_{n=1}^{\infty }\frac{\mu^{n}}{n!}\exp(-\mu )=1-\exp(-\mu )$$ 20

where $\mu =\frac{4}{3}\pi (r_{c}^{3}-a^{3})[Q]N_{A}\times 10^{-27}$ , [Q] is in M, and *r*_*c*_ and *a* are in Å. Therefore, assuming perfectly efficient quenching and *P*_*o*_ probability (eq 20) of having at least one neighbor upon excitation, the probability of survival upon excitation is 1 – *P*_*o*_ = exp­(−μ). This gives *I*_modified_ = *I*exp­(−μ) and the following *I*_*o*_/*I* relation:

$$\frac{I_{o}}{I_{\mathrm{modified}}}=\frac{I_{o}}{I}\exp(\mu )$$ 21

This (eq 21) is referred to in Figure 6 as “Smo. + crowding”, i.e., the neighbor modification is accounting for crowding. As we can see, this best fits the data and indicates that its inclusion is necessary when the *k*_CC_ approaches infinity (instantaneous reaction). If an increased time-window for counting static quenching was to be introduced, in order to account for experimental time-resolution, one may further consider modifying this crowding factor with time-window and the *k*_rxn_(*r*) variables.

Smoluchowski theory proves to be quite robust and predictive of the simulation data if the reaction is completely efficient (*k*_CC_ → *∞*), and the crowding-static quenching effects are taken into account (see Figure 6). Alternatively, we see it clearly does not perform when *k*_CC_ is low and when interference is observed (see Figure 8).

The Szabo model^17^ accounts for reactions within a given separation distance, *a* ≤ *r* ≤ *R*_react_, where a is the contact distance (usually just σ) and *R*_react_ is the reaction radius (i.e., 2^1/6^σ in our simulations). This provides a better comparison to soft-sphere models or short-distance-dependent processes, as it does not assume a reflecting boundary condition (like the CK model) and accounts for the range of distances the fluorophore and quencher can occupy while in contact. This best fits our data (see Figure 8) when a *k*_rxn_^*A*^(*r*) is implemented to quench with a specific probability *k*_CC_ within a given distance, also including the “crowding” quenching component introduced and used from Eads et al.^48^ The corresponding *k*_act_ (eq 22) as described by Szabo is as follows:

$$k_{\mathrm{act}}=4\pi D_{o}R_{\mathrm{react}}\frac{\epsilon \lambda \cosh(\lambda )-[\epsilon -(\lambda^{2}aR_{\mathrm{react}}/\epsilon )]\sinh(\lambda )}{a\lambda \cosh(\lambda )+\epsilon \sinh(\lambda )}$$ 22

where τ_CC_ = (*k*_CC_)^−1^, $\lambda =\epsilon /\sqrt{D_{o}\tau_{\mathrm{CC}}}$ , and ϵ = *R*_react_ – *a*. Given that *k*_CC_ is the rate of quenching while the quencher is in the vicinity, ϵ, of the fluorophore. This *k*_act_ can then be inserted into the standard Smoluchowski *I*_*o*_/*I* calculation, just as the CK *k*_act_ was, via $R_{\mathrm{eff}}=\frac{R_{\mathrm{FQ}}k_{\mathrm{act}}}{k_{\mathrm{act}}+4\pi R_{\mathrm{FQ}}D_{o}}$ . Note that the Szabo model does not converge to the Smoluchowski *I*_*o*_/*I* if *k*_CC_ → *∞*, indicating limitations for model equivalence.

Realistically, chemical quenching processes will follow some sort of distance-dependent ET reaction rate that may resemble a step function rate *k*_rxn_^*A*^(*r*) value if the β value for the *k*_rxn_^*B*^(*r*) is such that they have similar probability areas at that distance. Therefore, the Collins–Kimball and Szabo models may be tuned to fit experimental data with inefficient quenching and provide insight if the *k*_rxn_^*B*^(*r*) can be approximated by a fixed distance *k*_rxn_^*A*^(*r*). In cases where this does not hold, and when specific distance-dependent reaction processes are present, simulation provides an advantage to purely theoretical investigation. Alternatively, as seen in the simulations, a 1 Å^–1^ β value fits the step-function cutoff data quite well on the SV-plot, suggesting the step function can serve as a proxy for distance-dependence given the system’s distant-dependent rate has a comparable probable quenching volume.

For distance-dependent quenching models, the novelty of this simulation approach is demonstrated, allowing for the incorporation of nonstandard quenching criterion, which in this case is fluorescence quenching statistics across different β distance decay values. This may in turn incorporate yet more complex distant-dependent rates should the simulation of such inputs be desired.

PDE methods that implement diffusion and distance dependence quenching may also be used in order to extract theoretical fluorescence decay curves and fluorescence intensity. These are solved via the Crank–Nicolson method and trapezoidal integration.^39–42^ The theory entails *U*(*r*, *t*) as the survival probability of a Q–F* pair at time t, which originally were separated by distance *r* and may undergo distance-dependent quenching, with *k*(*r*) = *A*exp­(−β­(*r* – *r*_*o*_)). The PDE (eq 23) is solved to find *U*(*r*, *t*) and used to compute the total survival probability (eq 24), *P*(*t*), which is analogous to the fluorescence decay curve.

$$\frac{\partial U(r,t)}{\partial t}=D_{o}\left[\frac{\partial^{2}}{\partial r^{2}}+\frac{2\partial }{r\partial r}\right]U(r,t)-k(r)U(r,t)$$ 23

$$P(t)=\exp\left(-t/\tau_{o}-4\pi [Q]\int_{d}^{\infty }(1-U(r,t))r^{2}dr\right)$$ 24

The initial and boundary conditions are given as *U*(*r*, 0) = 1, (∂_*r*_ *U*(*r*, *t*))_*r*=*d*_ = 0, *U*(*∞*, *t*) = 1. The set of equations may then be iterated and solved by the tridiagonal matrix method.^55^ The SV-plot is then calculated from *I*_*o*_/*I* = τ_*o*_/∫_0_^*∞*^ *P*(*t*)*dt*. Overall, the fluorescence intensity results from the solved PDE differ quite significantly compared with the Langevin Dynamics simulation results produced. The comparison to our data matched better at the lower β values but even then did not begin to overlap our data point until very high quencher concentrations (ca. 500 mM), as shown in Figure 13. This demonstrates the importance and advantage of simulating the diffusional distance-dependent quenching explicitly, as in our model. Our method of explicit simulation thus provides the opportunity for other theories to be tested and validated.

**Figure 13:**
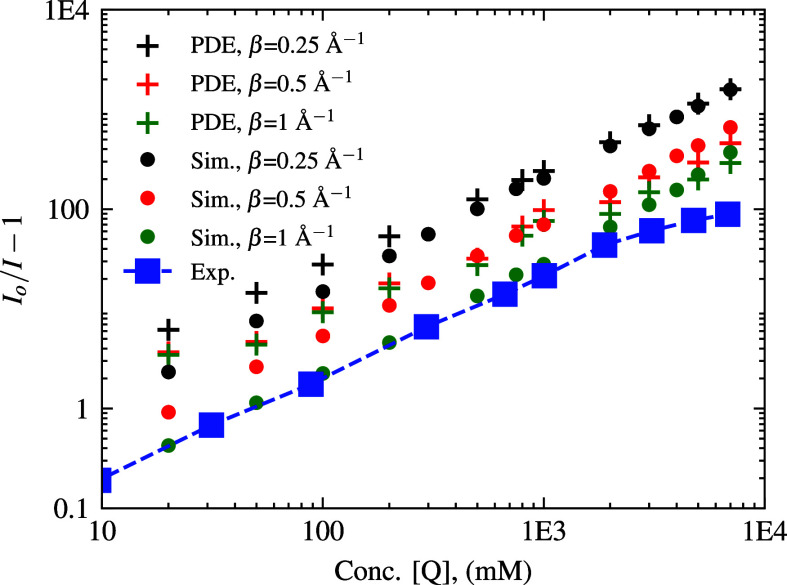
Log–log SV-plot of *I*_*o*_/*I* – 1 vs quencher concentration for simulated PDE data points. Langevin Dynamics simulations are shown for comparison. The PDE was solved with *D* = 0.35 Å^2^/ps and *A* = 0.02 ps^–1^, for *k*(*r*) = *A*exp­(−β­(*r* – *r*_*o*_)) at various β values. The experimental results are plotted with blue squares.

## 4 Conclusions

We have simulated bimolecular quenching systems via Langevin dynamics, extracting quenching rates and fluorescence intensity and subsequently comparing the resulting metrics to experiment. The molecular property inputs and spatiotemporal resolution of the simulation provide unique details such as quencher-fluorophore contact numbers, diffusion properties, nonequilibrium time-dependent RDFs, and transient effects. With increasing concentrations, different force fields, Coulombic effects, quenching efficiency, distance-dependent quenching, and molecular sizes, we see varying degrees of effect on the observed fluorescence decay curves, RDFs, *P*_static_, *P*_dynamic_, and *P*_intrinsic_ fractions, and *I*_*o*_/*I* – 1 plots.

Although classical SV theory and analysis work well in dilute regimes, if systems of interest begin to have higher quencher concentrations, an understanding of SV limitations and alternatives is required for physical interpretation. Investigation of high-quencher concentration regimes shows significant differences between simulations and classical SV theory. We find that in classical Stern–Volmer theory, $I=\frac{k_{f}F^{*}(0)}{k_{o}+k_{q}[Q]}$ is insufficient to describe higher concentration dynamics as it does not account for transient or static quenching effects, especially because at concentrations beyond the diffusion regime every fluorophore will have at least one quencher within quenching vicinity.

Important model limitations should be taken into account when evaluating the simulation results. Real systems are anisotropic and should account for orientation and atomistic detail. If an attractive force field is introduced, then there is a significant decrease in excited-state lifetimes. Additionally, the static component is approximated based on the probability of having one or more neighbor, though this will not necessarily be uniformly distributed in all systems. The observed static component (initial immediate quenching upon excitation) will also be largely dependent on the time-resolution of experiment and should be incorporated in models accordingly. Notably, quenching efficiency is not clearly accounted for in the theoretical models; in reality, this is distance-dependent, and multiple quenchers may have interference with one another. Additionally, accurate charge and electron transfer, hydrodynamic effects, and solvation free energy are also unaccounted for at very high concentrations.^14^

The presented methodology allows for the complex spectrum of quenching kinetics and dynamics to be evaluated across a variety of parameter combinations, giving insight into the importance and effect of each input. This simulation framework provides a base for further investigation to be performed on more complex systems, such as simulating systems with multiples species, activated quenching processes, complex formation, charge dynamics, distance-dependent electron transfer, and so forth. Extensions that balance the coarse-grained properties of the molecules and implicit solvation, with additional relevant atomistic detail and electron-transfer dynamics, may provide much sought after insight into solvent caging and charge escape dynamics which are relevant for solar fuel and energy extraction processes. Further method development and investigation into more complex quenching processes, allowing for multiple species, rates, and distance-dependent reaction functions to be included, are logical additions for further research.

## Supplementary Material



## Notes

Additional simulation results, analysis, graphics, and methods (PDF)
