# Supplementary material for: Reaction-Diffusion Dynamics Simulations of Bimolecular Quenching in Solution
Source: J Chem Theory Comput. 2026 Mar 17;22(7):3153–66. doi: 10.1021/acs.jctc.5c02114 (PMC13085250; doi:10.1021/acs.jctc.5c02114)
Supplement: Supplementary file 1 [file ct5c02114_si_001.pdf]

Supporting Information:

Reaction-Diffusion Dynamics Simulations of Bimolecular Quenching in Solution

Simon A. Liedtke, Martin Trulsson, and Petter Persson*

Division of Computational Chemistry, Department of Chemistry, Lund University, Box 124, SE-22100 Lund, Sweden

E-mail: petter.persson@compchem.lu.se

Contents

**Quenching rate and Taylor approximation**	**S2**
**Fitting**	**S4**
**Molar fractions**	**S6**
**Langevin Dynamics, Brownian motion**	**S6**
**Wolf Potential validation**	**S8**
**Radial Distribution Functions**	**S10**
**Neighbors and quenching modes**	**S12**
**Sensitivity of Intensity to Rate and LJ Attraction**	**S15**
**Visualization**	**S17**

Quenching rate and Taylor approximation

The probability of being quenched for a given fluorophore with one quencher within quenching distance, for the simple step-wise cutoff probability of reaction $k_{\text{rxn}}^{A}(r) = k_{\text{CC}}\Theta(R_{\text{react}} - r)$,

is:

$$P_{\text{quench}}(\delta t) = 1 - e^{-k_{CC}\delta t} \quad \text{(S1)}$$

Where δt is the time spent within the limit $r < 2^{1/6}\sigma$. r is the center-to-center distance between a fluorophore and a quencher.

Thus, the probability (eq S1) of quenching over a contact time δt can be displayed, as in Figure S1.

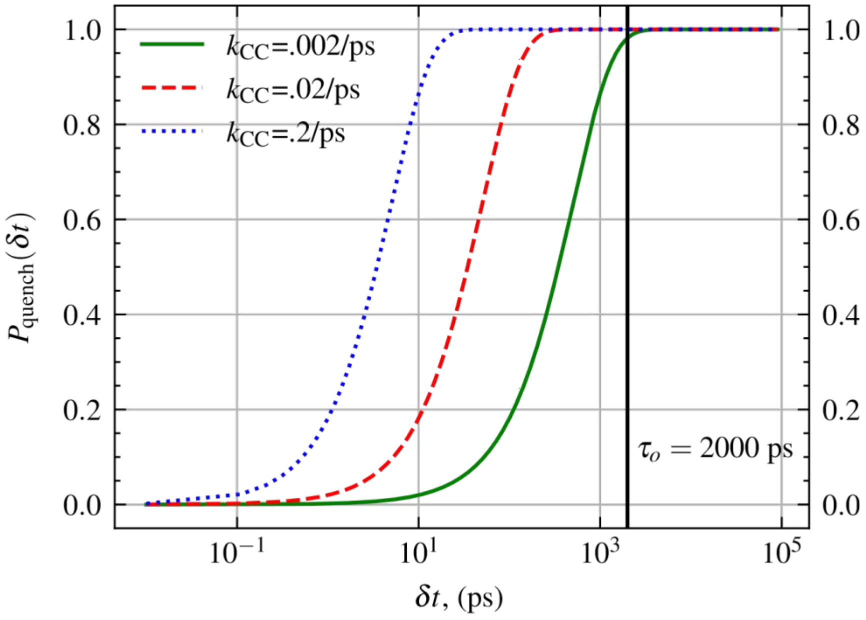

Figure S1: The probability of being quenched, $P_{\text{quench}}(\delta t)$, for a given fluorophore with one quencher within quenching distance over a time period δt. Assuming $k_{\text{rxn}}{}^{A}$ (r), and showing k_{CC} = 0.002,0.02, and 0.2 ps^{-1}. The intrinsic deactivation rate of the Fe(III) complex used in simulation, τ_o = 2 ns, is shown as reference. Plotted on semi-log scale, with x-log (time).

We implemented the Taylor approximation for applying the rate of quenching, $k_{\text{rxn}}(r)$ (assuming r is fixed), in our simulation. The probability of quenching per timestep-length, δt,

and with the quenching rate, $k_{\mathrm{rxn}}(r)$, is formally $1 - e^{-k_{\mathrm{rxn}}(r)\delta t}$ for the exact corresponding probability. However, we Taylor approximate this as $k_{\mathrm{rxn}}(r)\delta t$ during simulation. The following eq S2 can aid in the intuition behind this probability per timestep:

$$P_{\mathrm{quench}}(\delta t) = 1 - P_{\mathrm{surv}}(\delta t) = 1 - e^{-k_{\mathrm{rxn}}(r)\delta t} \simeq k_{\mathrm{rxn}}(r)\delta t \qquad \text{(S2)}$$

Where $P_{\mathrm{quench}}(\delta t)$ as the probability of the excited fluorophore being quenched and $P_{\mathrm{surv}}(\delta t)$ is the probability of the excited fluorophore surviving, during timestep-length δt. r is assumed to be fixed.

If using a distance dependent ET type efficiency, $k^{B}_{\mathrm{rxn}}(r) = k_{\mathrm{CC}} \exp\Big(-\beta(r - r_o)\Big)$, we can compare the discrete probability of quenching per timestep, p^{o}_{rxn}, used in our Molecular Dynamics simulations to assess the validity of the Taylor approximation used. Formally, assuming r is fixed, the exact probability is $p^{o}_{\mathrm{rxn}} = 1 - \exp\Big(-k_{CC} \exp\Big(-\beta(r - \sigma)\Big) \times \delta t\Big)$. Then, using the Taylor approximation: $p^{o}_{\mathrm{rxn}} = k_{CC} \exp\Big(-\beta(r - \sigma)\Big) \times \delta t$. We demonstrate and validate the difference between these two probabilities across distance, see Figure S2a and S2b, using k_{CC} = 0.02 ps^{-1}, β = 1 ˚A^{-1}, σ = 9 ˚A, and δt = 1 fs (values common to our simulation). As can be seen from Figure S2a, the difference between the two methods in calculating p^{o}_{rxn} across the length scale of r is at most near 5 orders of magnitude smaller than the actual p^{o}_{rxn}. This percentage difference ($100 \times \frac{\mathrm{Taylor} - \mathrm{Exact}}{\mathrm{Exact}}$), as shown in Figure

S2b, is at most 0.0001 % when at contact (σ = 9 ˚A). Therefore, we may conclude that this Taylor approximation is sufficient at our 1 femtosecond timestep used in Molecular Dynamics simulations.

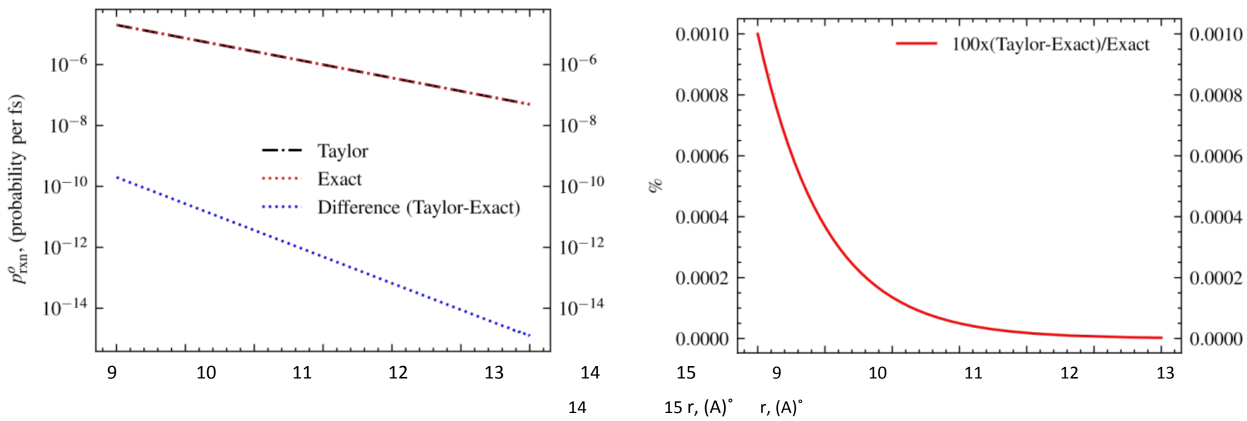

(a) (b)

Figure S2: (a) Plot of the $p^o{}_{rxn}$ across the length scale of r, from F-Q contact of 9 ˚A to 15 ˚A. The calculated Taylor approximation and the exact calculation for $p^o{}_{rxn}$ are shown. The difference between the Taylor approximation and exact calculation is also plotted. This is plotted with the semi-log (y-log) scale. k_{CC} = 0.02 ps^{-1}, β = 1 ˚A^{-1}, σ = 9 ˚A, and δt = 1 fs. (b) The percentage difference between the Taylor approximation and the exact calculation of $p^o{}_{rxn}$ across the length scale of r. Calculated as $\left(100 \times \frac{Taylor-Exact}{Exact}\right)$.

Fitting

Fitting the excited state fluorophore population over time (fluorescence decay curve) was examined by (log-fitting) the single exponential function: $A\exp\Big(-t(k_o + k[Q])\Big)$ (A and k as free variables), (log-fitting) the Smoluchowski[1] equation: $A\exp\Big(-t(k_o + k[Q] + \frac{B[Q]}{\sqrt{t}})\Big)$ (A, B, and k as free variables), and the two-exponential function (eq S3). Smoluchowski decay fitting performed better than single exponential fitting, but was still insufficient across the full set of concentrations relative to the two-exponential function (see Figure S3). The twoexponential function is what we used in practice for integration (using integration function in SciPy package with Python) when the full fluorescence decay curve was not available for fluorescence intensity, I, calculation. The two-exponential function is formulated with short-time and long-time components, and a fitting of A for the ratio contribution of each component.

$$F^*(t) = F^*(0^+)(1 - P_{\text{static}})^{\text{h}} A\exp[-t(k_o + k_{q1}[Q])] + (1 - A)\exp[-t(k_o + k_{q2}[Q])]^{\text{i}} \quad \text{(S3)}$$

Here, $0 < A < 1$, all k-values are positive, and the intrinsic rate k_o is incorporated in both exponentials. We assume $t - t_o$ is the starting point in fitting. P_{static} is fraction of excited fluorophore quenched by close-contact quencher upon excitation, this will be 0 when $k_{CC} \neq \infty$. $F^*(0^+)$ indicates the initial excited state fluorophore population excited, before close-contact static quenching upon excitation is taken into account.

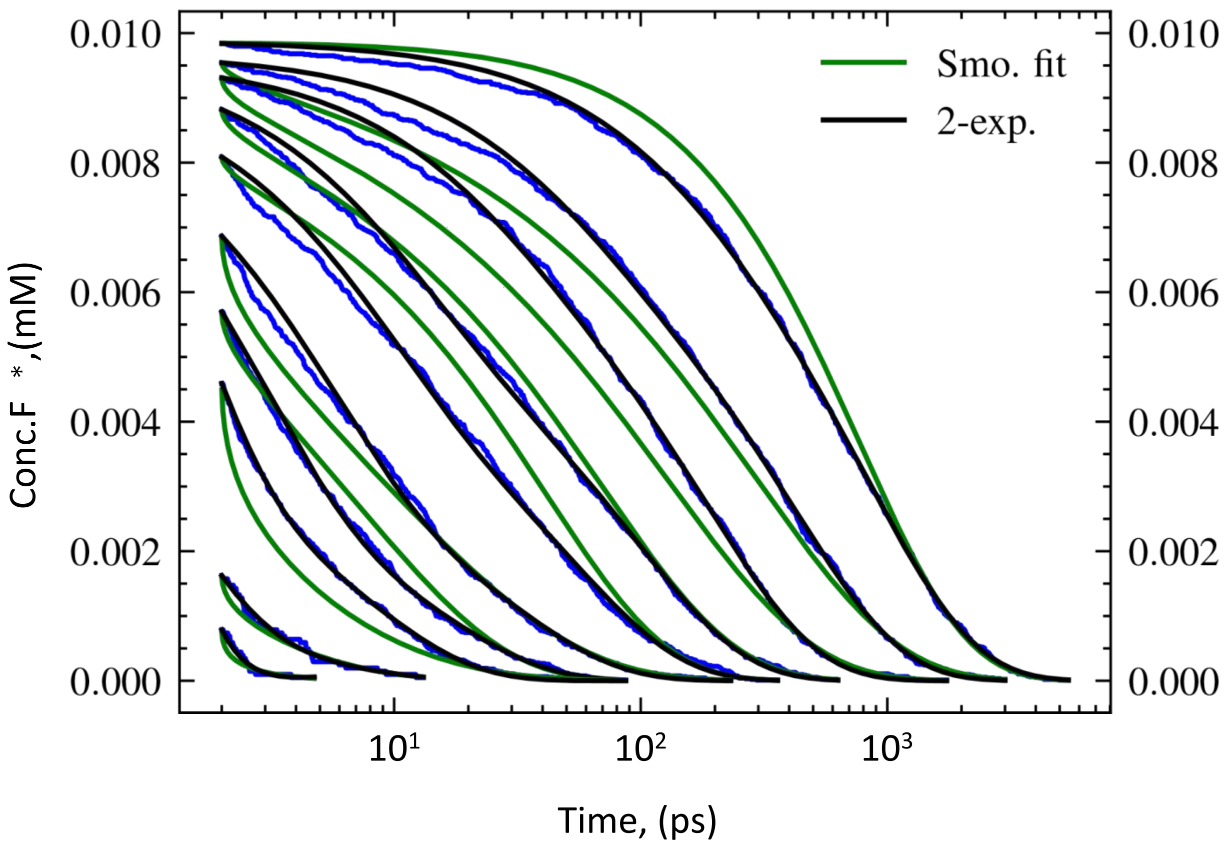

Figure S3: F^* fluorescence decay curves for quencher concentrations of 20, 50, 100, 200, 300, 500, 750, 1000, 2000, and 3000 mM. The step-wise cutoff probability of reaction was used $k^A_{\text{rxn}}(r)$ with $k_{\text{CC}} \rightarrow \infty$ (diffusion limited). Fits shown are the Smoluchowski fitting (Smo. fit) in green and the two-exponential fit (2-exp.) in black. 10 simulations were combined for each curve. Plotted on semi-log scale, with x-log (time).

Molar fractions

Repeated from the main article:

"A pure TEA solution with a density of 726 kg/m^3 corresponds to a concentration of 7.175 M. Similarly, for pure ACN, a mass of 41.05 g/mol and density of 786 kg/m^3 corresponds to a maximum concentration of 19.147 M. We modify the viscosity of our simulations, such that the diffusion values will gradually approach the values which are expected in a pure TEA (quencher) solution. A simple Arrhenius mixing law[2] for liquid mixtures was used: $\ln(\eta_{\text{mix}}) = \Sigma_i x_i \ln(\eta_i)$. This gives mixed viscosity (η_{mix}), which approaches that of TEA (η_{TEA} = 0.363 mPa · s) from that of ACN (η_{ACN} = 0.343 mPa · s), based on their molar fractions x_i (eq S4 and eq S5)such that, $\ln(\eta_{\text{mix}}) = x_{TEA} \ln(\eta_{\text{TEA}}) + x_{\text{ACN}} \ln(\eta_{\text{ACN}})$. The

calculated η_{mix} is used to solve the desired mutual diffusion, $D_{\text{aim}} = k_B T \left(\frac{1}{6\pi R_Q} + \frac{1}{6\pi R_F}\right)\frac{1}{\eta_{\text{mix}}}$, at each quencher concentration [Q]."

The molar fractions were calculated as follows:

$$x_{\text{ACN}} = \frac{\frac{(7.175M-[Q])}{7.175M}19.147M}{[Q] + \frac{(7.175M-[Q])}{7.175M}19.147M} \quad \text{(S4)}$$

$$x_{\text{TEA}} = \frac{[Q]}{[Q] + \frac{(7.175M-[Q])}{7.175M}19.147M} \quad \text{(S5)}$$

Langevin Dynamics, Brownian motion

Our simulations return the expected canonical Maxwell-Boltzmann velocity distributions (see Figure S4.), according to $f(v) \propto \exp\left[-\frac{mv^2}{2k_BT}\right]$. And the average speed will return as $\langle v \rangle = \sqrt{8k_BT/m\pi}$. This is as predicted, and due to our use of the Langevin dynamics thermostat.

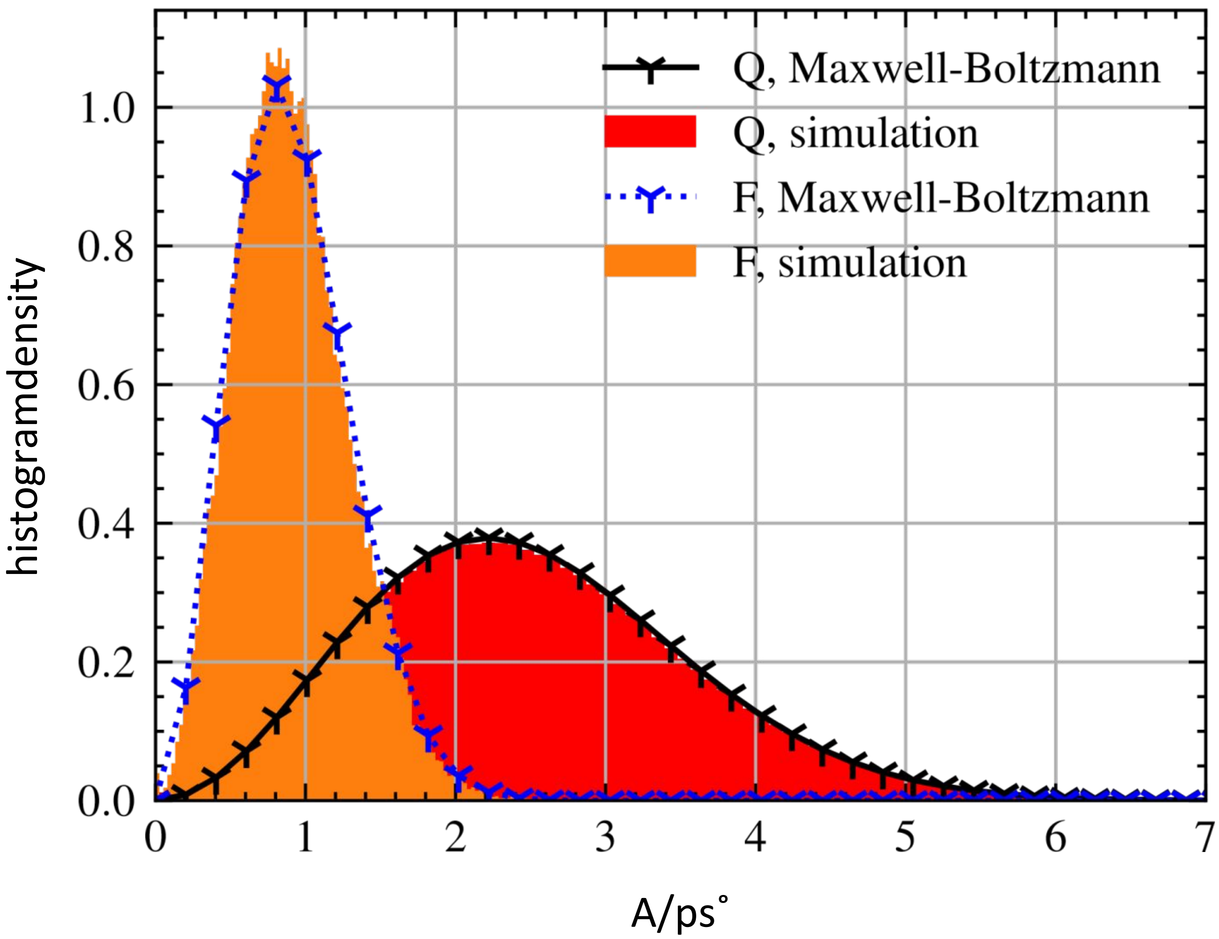

Figure S4: The canonical Maxwell-Boltzmann velocity distributions are shown for the fluorophore (F) in blue (simulation data in orange) and the quencher (Q) in black (simulation data in red). T = 298 K, m = 750.1 and 101.9 g/mol for the fluorophore and quencher respectively.

The Debye model of rotational Brownian motion,[3] gives the the molecular re-orientation time for a spherical molecule as: $\tau_{\text{rot}} = \frac{\eta V}{k_B T}$. Where V is the volume of the sphere.[3] This gives the fluorophore rotational time is τ_{rot} = 95.901 ps, which gives ≈ 0.0104 full rotations per ps. This is interestingly (although perhaps completely unrelated), of similar magnitude to the k_{CC} = 0.02 ps^{-1} we have used in simulation which matches the experimental data.

The VACF for each molecule type is theoretically calculated from the characteristic relaxation times.[3] $\langle v(0)v(t)\rangle = \langle v_o^2\rangle \exp(-\frac{t}{\tau_r}) = \frac{3k_BT}{m} \exp(-\frac{t}{\tau_r})$. Where $\tau_r = \frac{m}{6\pi\eta R}$ is the char-

acteristic relaxation time and $\langle v_o^2 \rangle = \frac{3k_BT}{m}$ is the mean square speed. The viscosity used is for acetonitrile, η_{ACN} = 0.343 mPa·s. We can observe this directly by extracting $\langle v(0)v(t)\rangle$ from our simulations. See Figure S5 for the comparison. The quencher characteristic relaxation time is $\tau_{Qr} = \frac{m_Q}{6\pi\eta R_Q} \approx 103.$9 fs and that of the fluorophore is $\tau_{Fr} = \frac{m_F}{6\pi\eta R_F} \approx 296.$4 fs.

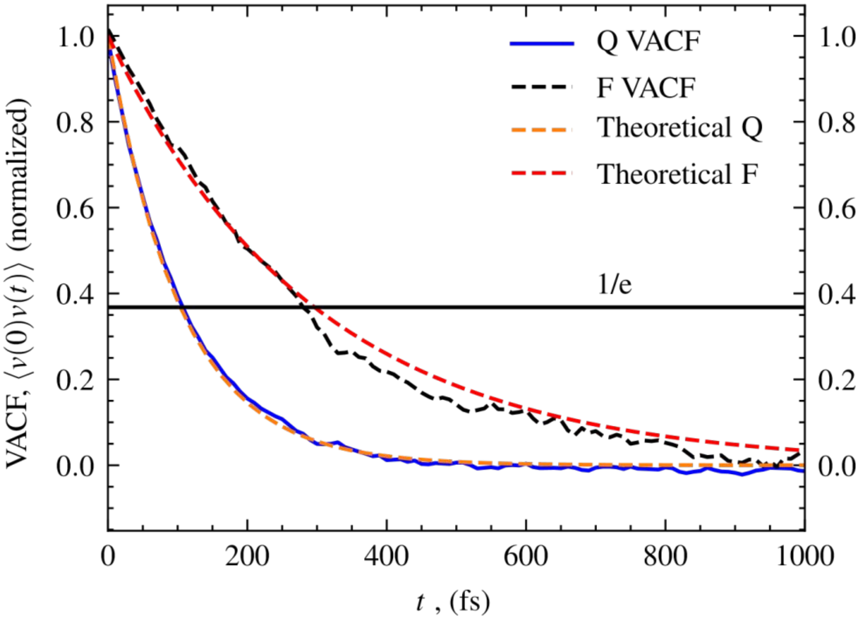

Figure S5: VACF, $\langle v(0)v(t)\rangle$, from the simulations and theoretical VACF as calculated from the characteristic relaxation times. Molecular characteristics (mass and radius) are the same as detailed in the paper. T=298 K.

Wolf Potential validation

We performed a force comparison, comparing the forces acting on the molecules in the simulation, in order to demonstrate the Wolf method[4] as viable approximation for simulating the coulomb force in our system. As can be seen Figure S6, the following force functions (eq S6, eq S7, and eq S8), using $F(r) = -\frac{dU(r)}{dr}$, are plotted:

The WCA force:[5]

$$F_{\mathrm{WCA}}(r) = 24\varepsilon(2\sigma^{12}r^{-13} - \sigma^{6}r^{-7})\Theta(2^{1/6}\sigma - r) \quad \text{(S6)}$$

Here, $\varepsilon = 1\ k_BT$, σ=9 ˚A, and $\Theta(x)$ is the Heaviside step-function.

The Coulomb force:

$$F_{\mathrm{Coulomb}}(r) = \frac{k_BT\lambda_B z_i z_j}{r^2} \quad \text{(S7)}$$

z_i and z_j are the valency of the respective ions (both assumed to be 1 here). λ_B is the Bjerrum length of acetonitrile: λ_B=15.25 ˚A.

The Wolf force:

$$F_{\mathrm{Wolf}}(r) = k_B T\lambda_B z_i z_j \quad \frac{\mathrm{erfc}(r\alpha)}{r^2} - \frac{\mathrm{erfc}(R_c\alpha)}{R_c^2} + \frac{2\alpha}{\sqrt{\pi}} \quad \frac{\exp(-\alpha^2 r^2)}{r} - \frac{\exp(-\alpha^2 R_c^2)}{R_c} \quad \text{(S8)}$$

As Figure S6 shows, the Coulomb and Wolf forces are much weaker (roughly an order of magnitude) than those of the WCA potential in general, up until the distance begins nearing the bottom of the LJ well ($r = 2^{1/6}\sigma$). Then, even on the log-log scale we see no visible departure between the Coulomb and Wolf force values until $r \approx 30$ ˚A. This indicates the Wolf potential is a valid replacement for standard Coulomb potential calculations, especially in our low ion density system.

R_c is the finite cutoff radius (implemented as box length L = 250˚A divided by 2, R_c = 125˚A), and $\alpha = 2/R_c$ is used as the complimentary error function damping parameter.

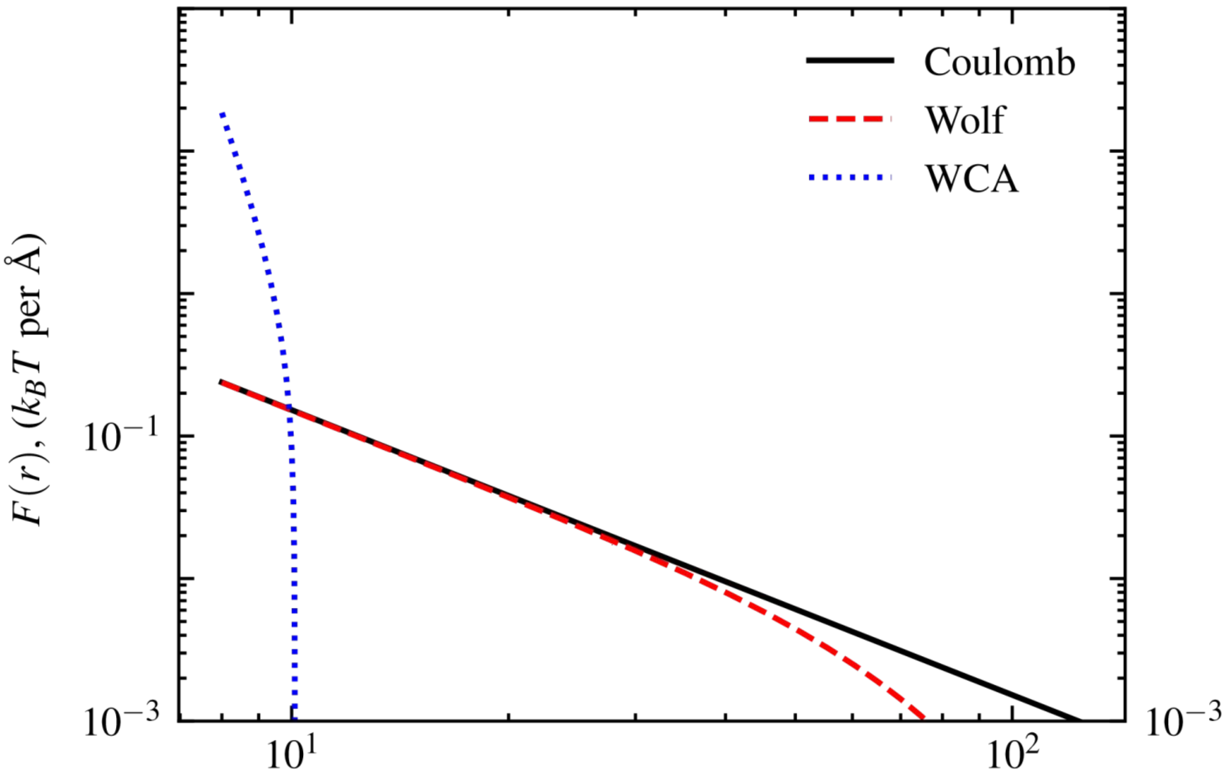

10^2 10^2

10^1 10^1 10^0 10^0

10^{-1}

10^{-2} 10^{-2}

r, (A)˚

Figure S6: Force (measured in $k_B T \cdot ^\circ A^{-1}$) comparison of the LJ potential, the Wolf potential, and standard Coulomb force. The range of r values shown is 8 to 125 ˚A. T=298 K. Plotted on the log-log scale.

Radial Distribution Functions

The Radial Distribution Functions (RDFs) for the fluorophore to all quenchers, $g_{FQ}(r)$, were calculated from the simulations. See Figure S7a to observe the RDF shape change across concentration from 0.1 M to the highest concentration of 7 M. See Figure S7b to observe the RDF shape change across concentration from 0.1 M to the highest concentration of 5 M. Both potentials were simulated with ε = 1 k_BT, and the LJ designated only had the LJ potential between fluorophore and quencher (Q-Q and F-F interactions still used only the WCA potential). It is clear from both plots that when higher concentrations are used, there is greater close packing and more sinusoidal shape to the RDF curve. It is also very clear from the two plots, that the LJ attractive potential plays a huge role in the shape of the curve and draws many quenchers into the neighbor region of the fluorophore; with the peak $g_{FQ}(r)$ value at 0.1 M [Q] in the LJ graph being even higher than the peak $g_{FQ}(r)$ value for 7 M [Q] in the WCA potential graph. Here we can clearly see why adding an attractive component to the potential can play a major role in quenching dynamics of the system, in almost all cases we would expect faster quenching rates.

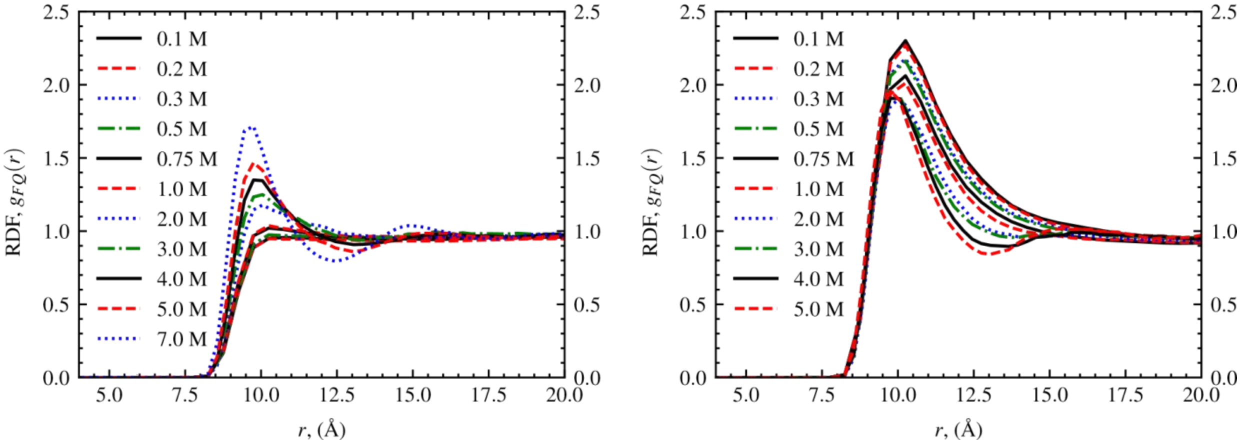

(a) WCA (b) LJ

Figure S7: (a) F-Q radial pair-correlation function $g_{FQ}(r)$, for the WCA potential system. Quencher concentrations shown, [Q], range from 0.1 to 7 M (b) F-Q radial pair-correlation function $g_{FQ}(r)$, for the LJ potential system (LJ is only for Q-F interactions, Q-Q and F-F interactions still used only the WCA potential). Quencher concentrations shown, [Q], range from 0.1 to 5 M.

A solution for $I_o/I - 1$ can also be computed by solely looking at the RDF integration and not accounting for diffusion, to be used for comparison (as seen in Figure S8). If the decay curve solely accounting for RDF reaction probability and intrinsic deactivation is expressed as: $F^*(t) = F_o^* \exp[-t(k_o + \int_0^\infty \rho g(r) A \exp(-\beta(r - r_o))4\pi r^2 dr)]$. Then, $I_o/I - 1 = \frac{\int_0^\infty \rho g(r) A \exp(-\beta(r-r_o))4\pi r^2 dr}{k_o}$. Where $\rho = N/V$ is the average number density. The results indicate this can give the general trend and magnitude, especially in the intermediate concentrations from 200-1000 mM, but it does not capture all reaction dynamics seen in the simulations; which is to be expected as it does not account for diffusion.

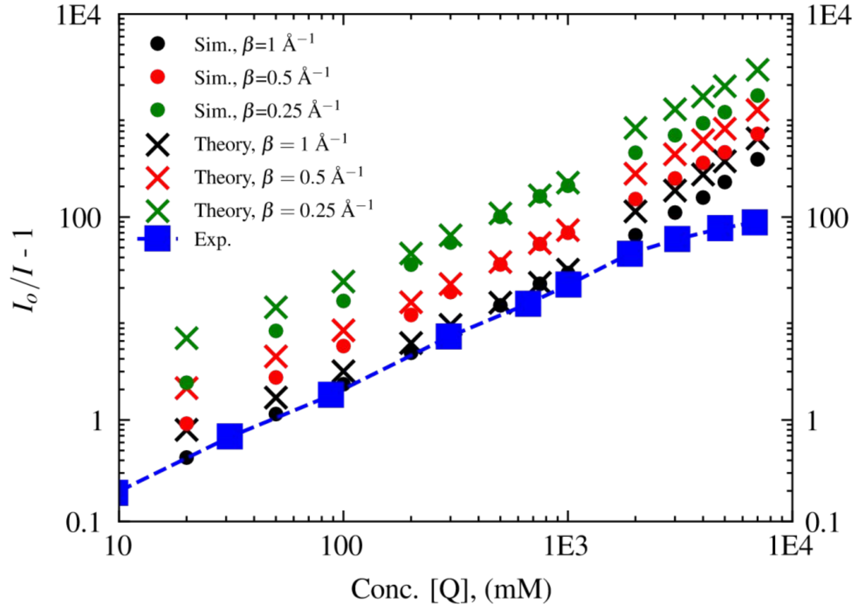

Figure S8: Log-log SV plot of I_o/I-1 vs quencher concentration for simulated $k^B_{\mathrm{rxn}}(r)$ type reactions, for various β and $k_{\mathrm{CC}} = 0.02$ ps^{-1}. Integrated RDF $I_o/I-1$ solutions, as described in text, also shown. The experimental comparison is plotted with blue squares.

Neighbors and quenching modes

The plots for fraction quenched across concentration for each mode of quenching (static, dynamic, and intrinsic) are displayed with plots for P_{static}, P_{dynamic}, and $P_{\text{intrinsic}}$ using various k_{CC} values between 0.02 and ∞ ps^{-1}.

The lower the rate of quenching, k_{CC}, the less static quenching has an effect. As can be seen in Figure S9a. This scales relatively linearly because it is theoretically only reliant on the average number of quencher neighbors for each fluorophore. Perhaps this could also be expanded to a set time-period after excitation, to catch more 'static' quenching which could be present in experimental measurement; though for our simulation we only monitor immediate quenching at the timestep after excitation.

Consequently, a lower k_{CC} results in more dynamic quenching (P_{dynamic}), due to a lower P_{static} contribution, see Figure S9b. Although, at lower concentrations (≤ 200 mM) and low k_{CC} (≈ 0.02 ps^{-1}), when there is room for diffusion away before a quenching event may occur, there is clear competition with $P_{\text{intrinsic}}$ (See Figure S9b and S9c). The $P_{\text{intrinsic}}$ is otherwise, unsurprisingly, directly influenced by the efficiency of k_{CC}; as a lower amount of quenching provides room for intrinsic deactivation.

Additionally displayed is the average number of quencher neighbors (Z_{FQ}) within $r = 2^{1/6}\sigma$ of each fluorophore. This is displayed across all concentrations of quenchers. This provides insight for likelihood of static quenching (by neighbors). See Figure S9d.

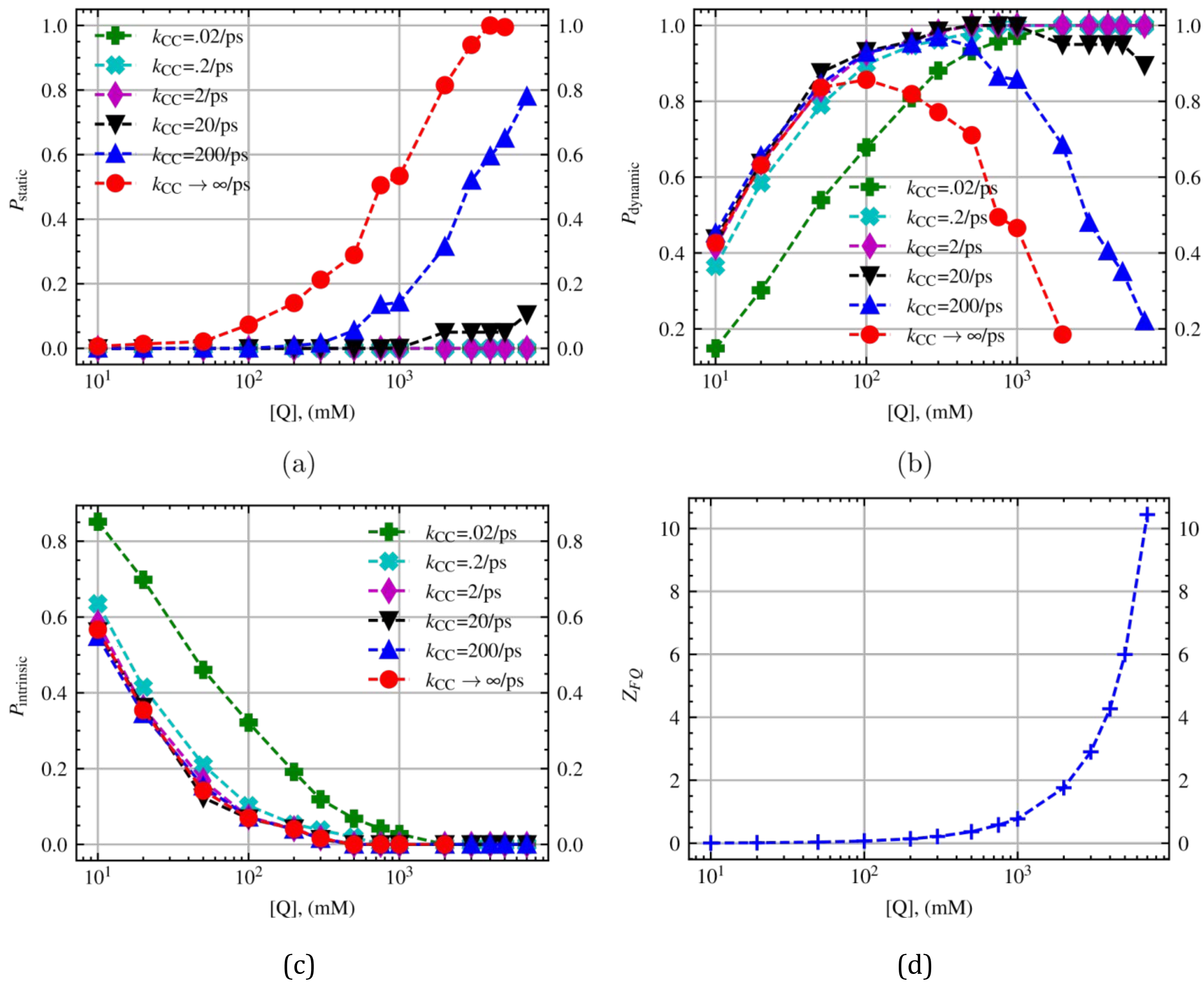

Figure S9: (a) P_{static} for various k_{CC} between 0.02 and ∞ ps^{-1}, versus quencher concentration. (b) P_{dynamic} for various k_{CC} between 0.02 and ∞ ps^{-1}, versus quencher concentration. (c) $P_{\text{intrinsic}}$ for various k_{CC} between 0.02 and ∞ ps^{-1}, versus quencher concentration. (d) Average number of quencher neighbors within $r = 2^{1/6}\sigma$ of a fluorophore (Z_{FQ}) versus concentration of quenchers. All graphs plotted on semi-log scale, with x-log (concentration).
Dashed lines are guides for the eyes.

Just as Figure S9d shows the average neighbors, we may also monitor the distribution of all quencher neighbors within $r = 2^{1/6\sigma}$ of each fluorophore in a simulation. See Figure S10 for a visualization of such a neighbor distribution histogram for 2, 3, 4, and 5 M of quenchers. As expected, the histogram is wider and shorter and also has a higher average neighbor count with increasing concentration.

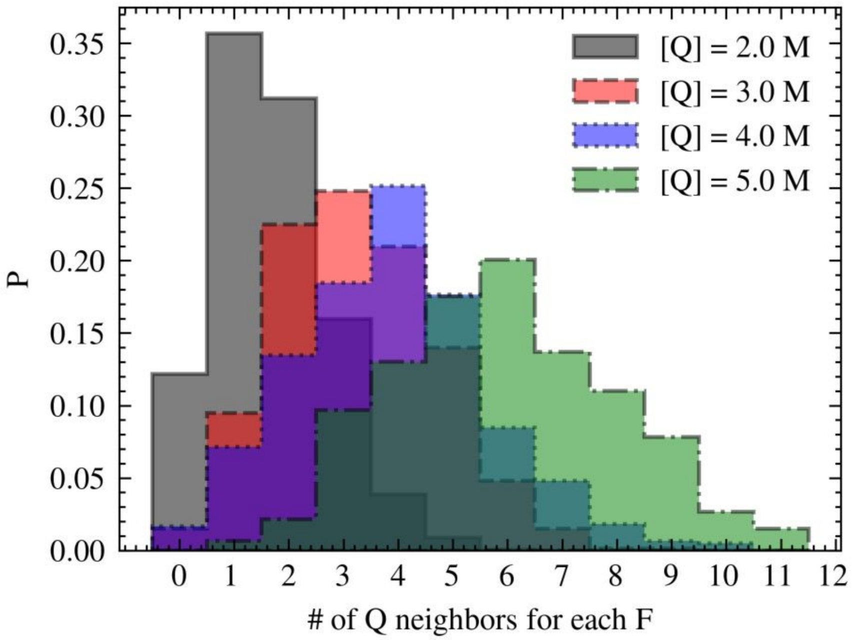

Figure S10: The histogram distribution of all quencher neighbors within r = 1.26σ of each fluorophore in a simulation. Shown for 2, 3, 4, and 5 M quencher concentrations.

As can be seen, and may be expected, these fluorophore-quencher neighbor distribution histograms appear to follow a Poisson distribution. Figure S11 plots the fluorophorequencher neighbor distributions for 0.75 to 5 M and the corresponding calculated probability mass functions (pmf) of the Poisson distribution. Where the Poisson pmf is $f(k, \mu) = \frac{\mu^k e^{-\mu}}{k!}$, for μ average neighbors (Z_{FQ}) and k is the number of neighbors in this case.

We display the pmf calculated from the Z_{FQ} retrieved in simulation, and also the pmf with the Z_{FQ} predicted solely by the volume of the neighbor area (R_{out}^3 - R_{in}^3) around the fluorophore and the quencher concentration [Q]. Where the outer volume radius from center R_{out} = $2^{1/6}\sigma$ and the contact point a was estimated at 8.7 ˚A (slightly below σ). Predicted $Z_{FQ} = \frac{4}{3}\pi(R_{\text{out}}^3 - a^3)[\text{Q}]N_A \times 10^{-27}$, [Q] is in M, and r_c and a are in ˚A. This predicted volume pmf appears to be reasonably accurate but breaks down at the highest concentration of 5 M, likely due to more packing pressure from surrounding quencher layers.

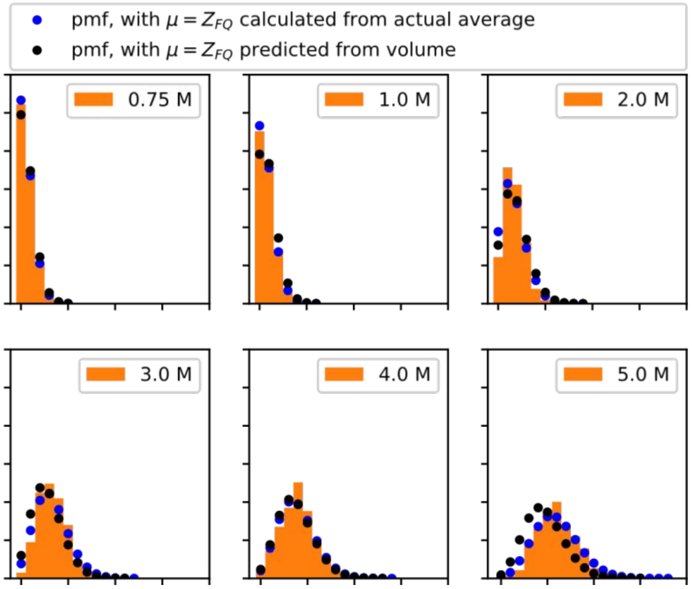

Figure S11: The histogram distribution of all quencher neighbors within $r = 2^{1/6}\sigma$ of each fluorophore in a simulation. Shown for 0.75, 1, 2, 3, 4, and 5 M quencher concentrations. Also displayed are the pmf calculated from the Z_{FQ} retrieved in simulation (blue dots) and the pmf with the Z_{FQ} predicted by the volume of the neighbor area around the fluorophore (black dots).

Sensitivity of Intensity to Rate and LJ Attraction

Simulations were run with varied quenching rates to assess the sensitivity the intensity, compared via $I_o/I - 1$, of the selected quenching rate parameter, k_{CC}, for step-wise cutoff reaction rates $k^{A}_{\mathrm{rxn}}(r)$. Results are shown in Figure S12 to compare k_{CC} values of 0.01, 0.02, and 0.05 ps^{-1}. As seen in the figures below, the results are as expected with a faster quenching rate (0.05 ps^{-1}) producing an upward shift in the $I_o/I - 1$ and a slower quenching rate (0.01 ps^{-1}) producing a downward shift relative to the 0.02 ps^{-1} rate used in the main paper. This general trend can be approximated by Equations 4 and 7 in the main text, where k_{CC} adjusts the linear slope ($k_q = \frac{k_{\mathrm{diff}}k_{\mathrm{CC}}}{k_{\mathrm{diff}} + k_{\mathrm{CC}}}$) of the classical SV equation: $\frac{I_o}{I} = 1 + \frac{k_q[Q]}{k_o}$. This trend can

also be directly predicted via the Szabo equation for $\frac{I_o}{I}$, eq 21 in the main text, with τ_{CC} = $(k_{CC})^{-1}$ being adjusted for the given k_{CC} values. As can be seen in Figure S12, this is highly predictive and simulation data only slightly departs at high quencher concentrations of 2M and above. As in the main text, the The experimental comparison is from Rosemann et al.[6]

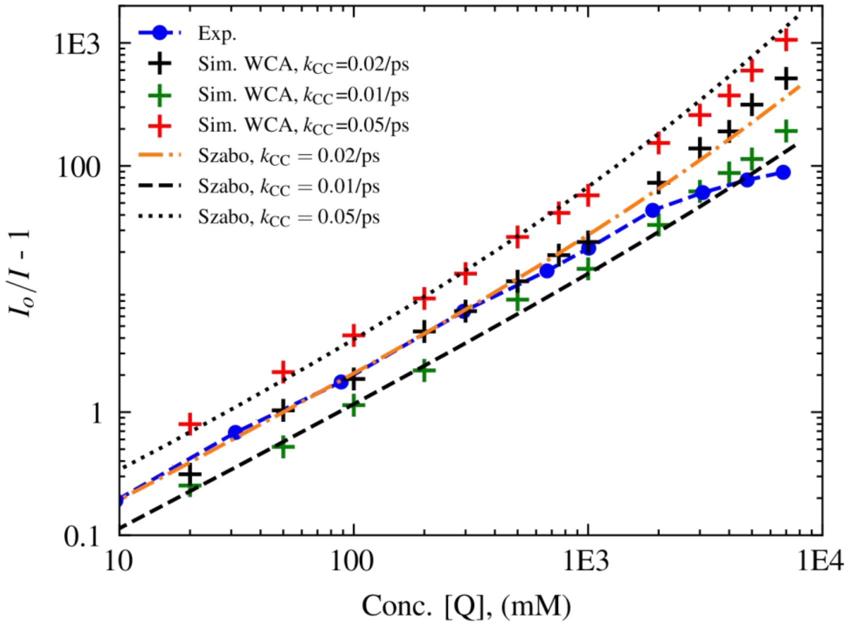

Figure S12: Log-log SV-plot of I_o/I-1 vs quencher concentration, when inefficient quenching is implemented. Simulations displayed for WCA potential with $k^A_{\mathrm{rxn}}(r)$ and k_{CC} = 0.01, 0.02, and 0.05 ps^{-1}. Theoretical curves incorporating k_{CC} in the Szabo model are also included. The experimental comparison is plotted with blue circles.

Also presented, in Figure S13, are results from step-wise cutoff reaction rates $k^A_{\mathrm{rxn}}(r)$using k_{CC}=0.02 ps^{-1} with the LJ potential, in comparison to the WCA potential, in order to asses the sensitivity to an attractive force-field component with inefficient quenching. An attractive LJ potential with a ε value of 1 k_BT was used and demonstrated increased rates of quenching (seen from the lower intensity I and thus higher shift in the SV plot) at the lower concentrations. This is expected, as the attractive component of the LJ potential well will increase fluorophore-quencher aggregation and time spent within quenching distance.

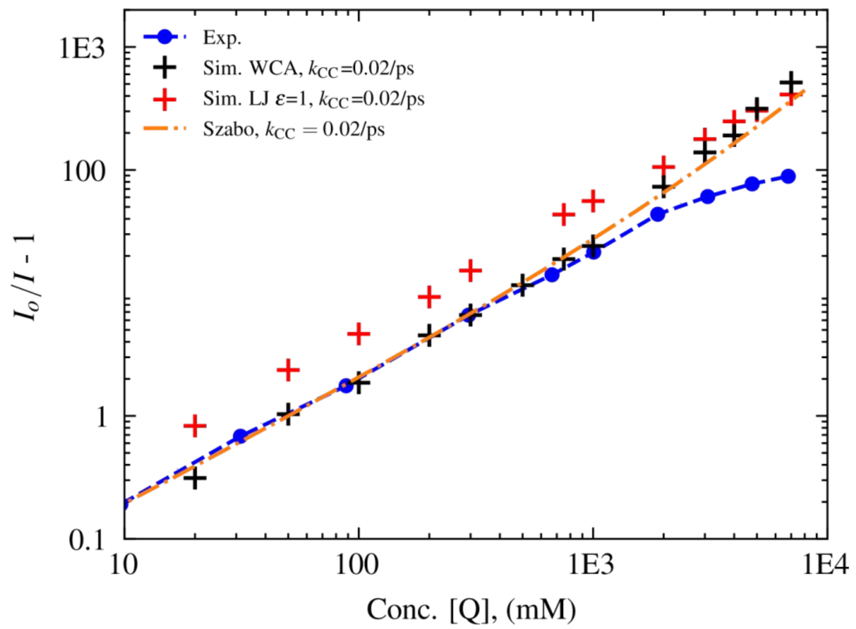

Figure S13: Log-log SV-plot of I_o/I-1 vs quencher concentration, when inefficient quenching is implemented. Simulations displayed for WCA potential and LJ potentials (ε = 1 and 3 k_BT), with $k^A_{\mathrm{rxn}}(r)$ and k_{CC} = 0.02 ps^{-1}. Theoretical curve incorporating k_{CC} in the Szabo model is also included. The experimental comparison is plotted with blue circles.

Visualization

Below we display the visualization of the simulation box, using the Ovito software. See Figures S14a, S14b, S14c, and S14d. The total fluorophore concentration is: [F] + [F*] = 10 mM. The number of excited state or ground state fluorophores will depend on the snapshot time (how many of the initial 10 mM F* have returned to the ground state F). The quencher concentrations, [Q], shown are 50, 200, 500, and 1000 mM. L= 250 ˚A.

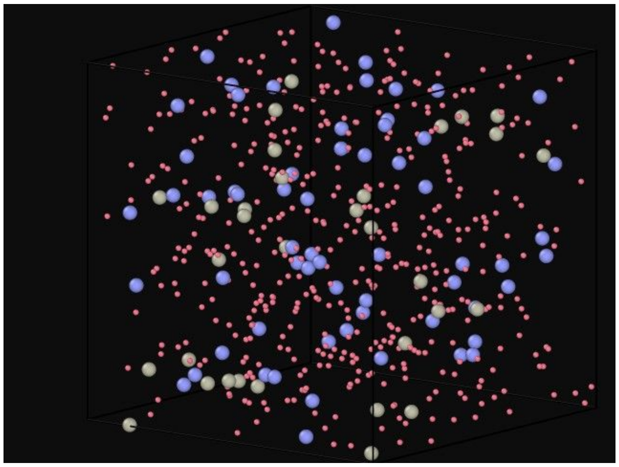

(a) [Q] = 50 mM

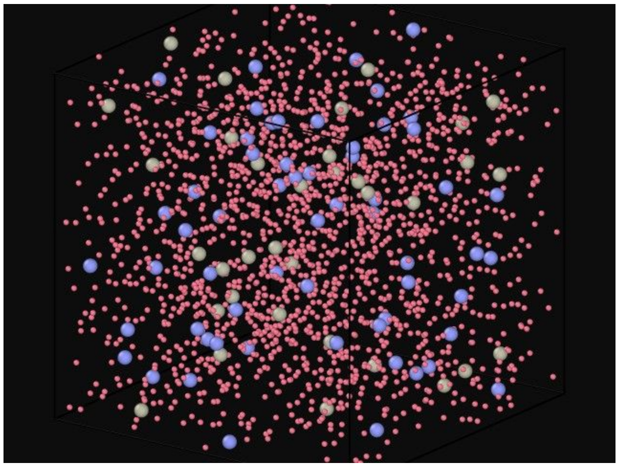

(b) [Q] = 200 mM

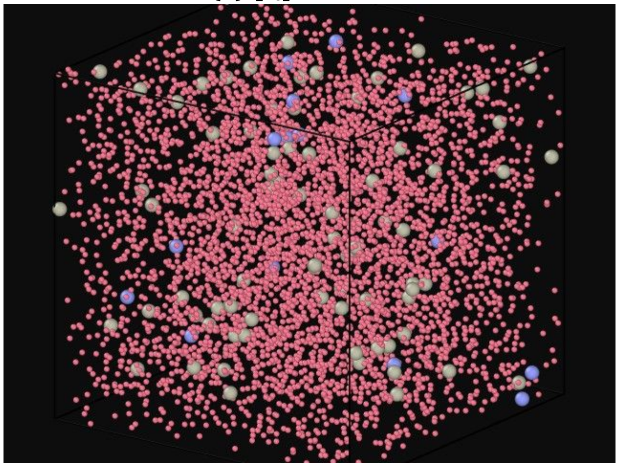

(c) [Q] = 500 mM

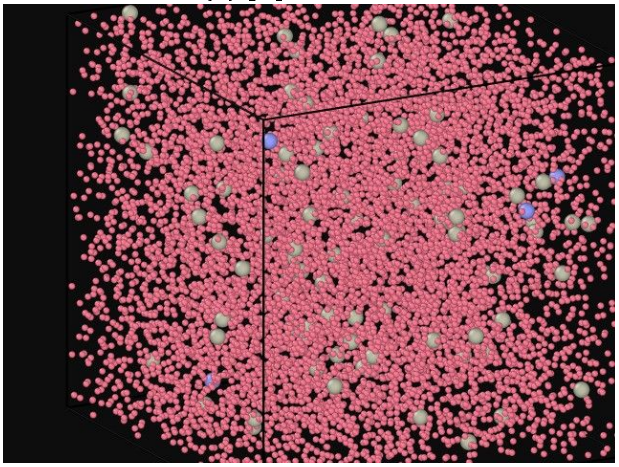

(d) [Q] = 1000 mM

Figure S14: 3D visualization of simulation box. Initial [F] is 10 mM for all. F is tan-gray, F* is light-blue, Q is light-red (a) 50 mM quencher concentration (b) 200 mM quencher concentration (c) 500 mM quencher concentration (d) 1000 mM quencher concentration

References

(1) Smoluchowski, M. v. Versuch einer mathematischen Theorie der Koagulationskinetik kolloider Lo¨sungen. *Zeitschrift fu¨r physikalische Chemie* **1918**, *92*, 129–168.

(2) Arrhenius, S. Uber die innere Reibung verdu¨nnter w¨asseriger Lo¨sungen.¨ *Zeitschrift fu¨r Physikalische Chemie* **1887**, *1*, 285–298.

(3) Rice, S. A. *Diffusion-limited reactions*; Elsevier, 1985.

(4) Wolf, D.; Keblinski, P.; Phillpot, S.; Eggebrecht, J. Exact method for the simulation of Coulombic systems by spherically truncated, pairwise r- 1 summation. *The Journal of chemical physics* **1999**, *110*, 8254–8282.

(5) Weeks, J. D.; Chandler, D.; Andersen, H. C. Role of repulsive forces in determining the equilibrium structure of simple liquids. *The Journal of chemical physics* **1971**, *54*, 5237–5247.

(6) Rosemann, N. W.; Ch´abera, P.; Prakash, O.; Kaufhold, S.; Wa¨rnmark, K.; Yartsev, A.; Persson, P. Tracing the Full Bimolecular Photocycle of Iron(III)–Carbene Light Harvesters in Electron-Donating Solvents. *J. Am. Chem. Soc.* **2020**, *142*, 8565–8569, PMID:
32307993.
